# Development of sustainable green geopolymer concrete incorporating sugarcane bagasse ash

**DOI:** 10.1038/s41598-026-63157-z

**Published:** 2026-07-28

**Authors:** Mehad Mahmoud, Sayed Salah, Yasmin Hefni Abdel Aziz, Taha A. El-Sayed

**Affiliations:** 1 Structural Engineering Department, Shoubra Faculty of Engineering, Benha University, Banha, Egypt; 2 Civil Engineering Department, Faculty of Engineering, Modern University for Technology & Information, Cairo, Egypt

**Keywords:** Alkaline activation, Elevated temperatures, Fly ash, Geopolymer concrete, Microstructure analysis, Silica fume, Sugarcane bagasse ash, Sustainability, Engineering, Environmental sciences, Materials science

## Abstract

Sugarcane bagasse ash (SCBA) is increasingly being considered as an alternative aluminosilicate source for geopolymer binders. however, limited studies have comprehensively evaluated the combined effects of high SCBA replacement levels, alkaline activator molarity, thermal performance, microstructural characteristics, and economic feasibility. Therefore, this study investigates the use of SCBA as a partial and near-complete replacement of the total binder in geopolymer concrete. Fifteen mixtures were prepared with SCBA replacement levels of 0, 25, 50, 75, and 95% at NaOH molarities of 12, 14, and 16 M. Fresh, mechanical, durability, thermal, and microstructural properties, along with cost analysis, were comprehensively evaluated. The mixture containing 25% SCBA and activated with 14 M NaOH showed the best overall performance, with a compressive strength of 30.13 MPa, a splitting tensile strength of 3.02 MPa, and a sorptivity of 2.449 × 10⁻³ g/cm·s⁰·⁵ at 28 days. After exposure to 200 °C, its compressive strength increased by 39.30%, before declining at higher temperatures. Mixtures with higher SCBA contents showed slower strength loss, suggesting improved thermal stability. SEM–EDX observations were consistent with these results, as the optimum mixture showed a dense geopolymeric matrix, while higher SCBA contents and elevated temperatures led to increased porosity and microcracking. The cost analysis also showed that production cost decreased considerably as the SCBA content increased. Overall, the results indicate that moderate SCBA replacement can provide a practical balance between mechanical performance, durability, thermal resistance, and cost, supporting the use of SCBA as a sustainable binder component in geopolymer concrete.

## Introduction

The construction industry is one of the major contributors to global carbon emissions due to the extensive use of ordinary Portland cement (OPC). Cement production accounts for approximately 5–8% of global anthropogenic CO₂ emissions because of the calcination process and the high energy demand associated with manufacturing. Consequently, considerable attention has been directed toward developing sustainable cement-free binders capable of reducing the environmental impact of construction materials while maintaining satisfactory engineering performance^1–4^.

Among the available alternatives, geopolymer binders have emerged as a promising binder system produced through the alkali activation of aluminosilicate-rich materials such as fly ash (FA), ground granulated blast furnace slag (GGBS), metakaolin, and agricultural ashes. The geopolymerization process leads to the formation of binding gels, including sodium aluminosilicate hydrate (N–A–S–H) and calcium aluminosilicate hydrate (C–A–S–H), which contribute to strength development, durability enhancement, and microstructural densification. As a result, geopolymer concrete represents a viable alternative to OPC-based concrete with a lower environmental footprint and potentially improved long-term performance^5–7^.

In addition to precursor composition, the performance of geopolymer concrete is strongly governed by the characteristics of the alkaline activator, supplementary silica sources, and curing conditions^8,9^. The incorporation of silica fume has been reported to enhance matrix densification, reduce porosity, and improve mechanical and durability properties by supplying additional reactive silica for geopolymer gel formation^10–14^. Similarly, increasing the molarity of alkaline activators within an appropriate range promotes the dissolution of aluminosilicate species and accelerates geopolymerization, resulting in improved strength and microstructural development^15–18^. Faisal and Muhammad^19^ demonstrated that geopolymer systems activated with NaOH and Na₂SiO₃ achieved their highest compressive strength at an SiO₂/Al₂O₃ ratio of 2.5, highlighting the significant influence of alkaline activation parameters on geopolymer development. Curing conditions in geopolymer concrete also play a critical role in determining geopolymer performance. Verma et al.^20^ reported that heat curing significantly enhances compressive strength, whereas Ali et al.^21^ demonstrated that Sugarcane bagasse ash -based geopolymer mixtures can achieve satisfactory mechanical performance under ambient curing conditions.

The evaluation of geopolymer concrete at elevated temperatures has also received increasing attention because of its potential use in fire-resistant structures. Previous studies have reported that geopolymer binders can retain a considerable proportion of their strength at moderate temperatures, mainly due to additional geopolymeric reactions, matrix densification, and internal moisture redistribution^21–26^. For example, Tanu et al.^22^ observed that GGBS–SCBA geopolymer concrete retained more than 50% of its compressive strength up to 600 °C, while Abed et al.^25^ report satisfactory performance up to 400 °C before significant degradation occurred. Beyond technical performance, industrial and agricultural by-products as geopolymer precursors contribute significantly to waste valorization, resource conservation, and cost savings ranging from 10.87% to 17.77% compared with conventional concrete^3–7,26^.

Among these by-products, sugarcane bagasse ash (SCBA) has gained considerable attention due to its silica-rich composition and environmental benefits. Several studies have investigated SCBA as a partial cement replacement in conventional concrete systems^27–29^, with Kumara et al.^27^ reporting optimum mechanical performance at a 15% replacement level. More recently, SCBA has been utilized as a supplementary silica-rich precursor in geopolymer systems^30–35^. Rihan et al.^33^ found that moderate SCBA contents (5–20%) enhanced strength development, while parallel studies reported improvements in mechanical and durability properties within similar replacement ranges^30–35^. Nevertheless, excessive SCBA incorporation generally reduces performance because of its porous structure, unburnt carbon content, and relatively low reactivity, which can hinder matrix densification and geopolymer gel formation^31–35^.

Despite growing interest in SCBA-based geopolymers, existing studies have primarily focused on low replacement levels, isolated performance characteristics, or specific curing regimes. Consequently, a comprehensive evaluation examining the concurrent influences of high-volume SCBA substitution, alkaline activator molarity, durability, elevated-temperature behavior, and microstructural evolution within a single experimental framework remains absent. In particular, the feasibility of replacing GPC total binders with SCBA at ultra-high levels—approaching near-complete substitution up to 95%—remains poorly understood. Furthermore, the coupled effects of such extreme SCBA content and activator molarity on thermal resistance, sorptivity, and microstructural stability have yet to be systematically addressed.

Therefore, the novelty of the present study lies in the comprehensive evaluation of geopolymer concrete incorporating ultra-high SCBA replacement levels ranging from 0% to 95% synthesized under varying sodium hydroxide )NaOH) concentrations (12 M, 14 M, and 16 M). This investigation uniquely integrates fresh-state properties, mechanical performance, long-term durability, thermal resistance, microstructural evolution, and cost analyses within a unified framework to determine the optimum mix design. By elucidating the fundamental mechanisms governing high-volume agricultural waste incorporation, these findings establish the technical viability of utilizing SCBA as a dominant geopolymeric precursor, thereby advancing the development of resource-efficient construction materials.

## Experimental work

A total of fifteen geopolymer concrete mixtures were prepared and divided into three groups according to NaOH molarity (12 M, 14 M, and 16 M), as presented in Table 1. Within each group, sugarcane bagasse ash (SCBA) was incorporated at 0%, 25%, 50%, 75%, and 95% of the total binder mass, while silica fume was maintained constant at 5%. SCBA progressively replaced fly ash; therefore, the A95, B95, and C95 mixtures contained 95% SCBA and 5% silica fume, with no fly ash, representing complete replacement of fly ash. Across all fifteen mixtures, the total alkaline activator content, silica fume, superplasticizer, and fine and coarse aggregate contents were kept constant to isolate the combined effects of SCBA content and NaOH molarity.

The specific selection of these mixture parameters was based on findings reported in previous studies. NaOH molarity levels of 12 M, 14 M, and 16 M were selected to evaluate the influence of alkaline concentration on geopolymerization and strength development^16–18^. The Na₂SiO₃/NaOH solution mass ratio was fixed at 2.5, as this ratio has been reported to provide a suitable balance between workability and mechanical performance^10,16,19^. In addition, silica fume was incorporated at 5% of the total binder mass to provide additional reactive silica and enhance matrix densification^11–14^. Heat curing at 70 °C for 24 h was adopted to accelerate geopolymerization and improve early-age strength development in the fly ash–SCBA geopolymer system^33,36^.

**Table 1:** Mix proportions of the geopolymer concrete mixtures) Kg per 1 m^3^) concrete.

Mix ID	Molarity	Alk.	S.F	F.A	SCBA	NaOH solution	Na_2_Sio_3_ solution	S.*P*	Sand	Dolomite
Control A	12 M	180	20	380	0	51.43	128.57	10	688	1100
A25		180	20	280	100	51.43	128.57	10	688	1100
A50		180	20	180	200	51.43	128.57	10	688	1100
A75		180	20	80	300	51.43	128.57	10	688	1100
A95		180	20	0	380	51.43	128.57	10	688	1100
Control B	14 M	180	20	380	0	51.43	128.57	10	688	1100
B25		180	20	280	100	51.43	128.57	10	688	1100
B50		180	20	180	200	51.43	128.57	10	688	1100
B75		180	20	80	300	51.43	128.57	10	688	1100
B95		180	20	0	380	51.43	128.57	10	688	1100
Control C	16 M	180	20	380	0	51.43	128.57	10	688	1100
C25		180	20	280	100	51.43	128.57	10	688	1100
C50		180	20	180	200	51.43	128.57	10	688	1100
C75		180	20	80	300	51.43	128.57	10	688	1100
C95		180	20	0	380	51.43	128.57	10	688	1100

The reported values represent the total masses of the alkaline solutions added to each mixture. Different NaOH molarities (12 M, 14 M, and 16 M) were obtained by varying the masses of NaOH flakes and distilled water while maintaining a constant total NaOH solution mass.

The percentages used in the mixture labels represent the SCBA content relative to the total binder mass. A95, B95, and C95 contain 95% SCBA and 5% silica fume, with no-fly ash.

**Fig. 1:**
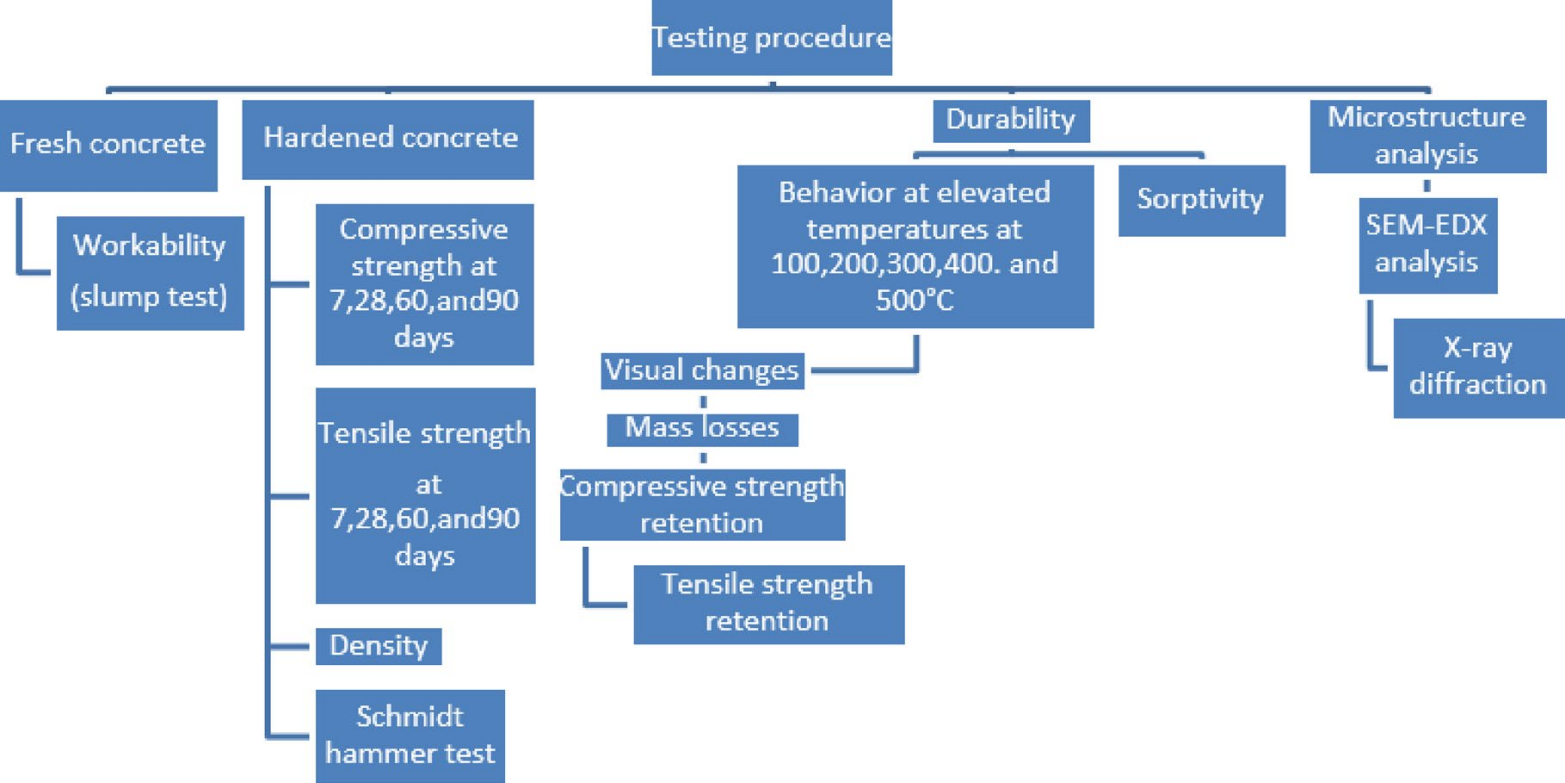
Experimental flowchart of the testing program.

Figure 1 illustrates the experimental flowchart of the testing program. All experimental investigations were conducted under controlled laboratory conditions and were further supported by microstructural characterization. The GPC mixtures were evaluated in both fresh and hardened states. Fresh properties were assessed using the slump test, while hardened properties included density, compressive strength, splitting tensile strength, and Schmidt hammer testing. Durability was evaluated through sorptivity and after exposure to elevated temperatures ranging from 100 °C to 500 °C for 2 h, followed by measurements of mass loss and residual mechanical properties. For each mixture, a total of 19 specimen groups—comprising 10 cubic and 9 cylindrical configurations—were prepared, with three identical specimens tested for each testing condition to ensure reproducibility. Accordingly, the reported results represent the average of these three measurements. Microstructural characterization was conducted using XRD, SEM, and EDX analyses to investigate the effects of SCBA content, NaOH molarity, and elevated temperature on the material microstructure.

### Materials

#### Fly ash

**Fig. 2:**
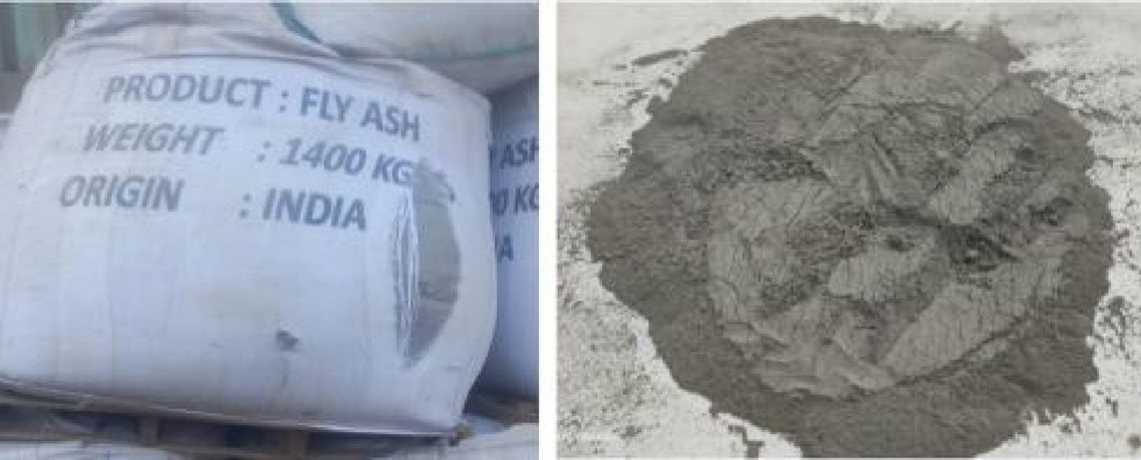
Visual appearance of the fly ash powder used as the primary geopolymeric precursor.

**Fig. 3:**
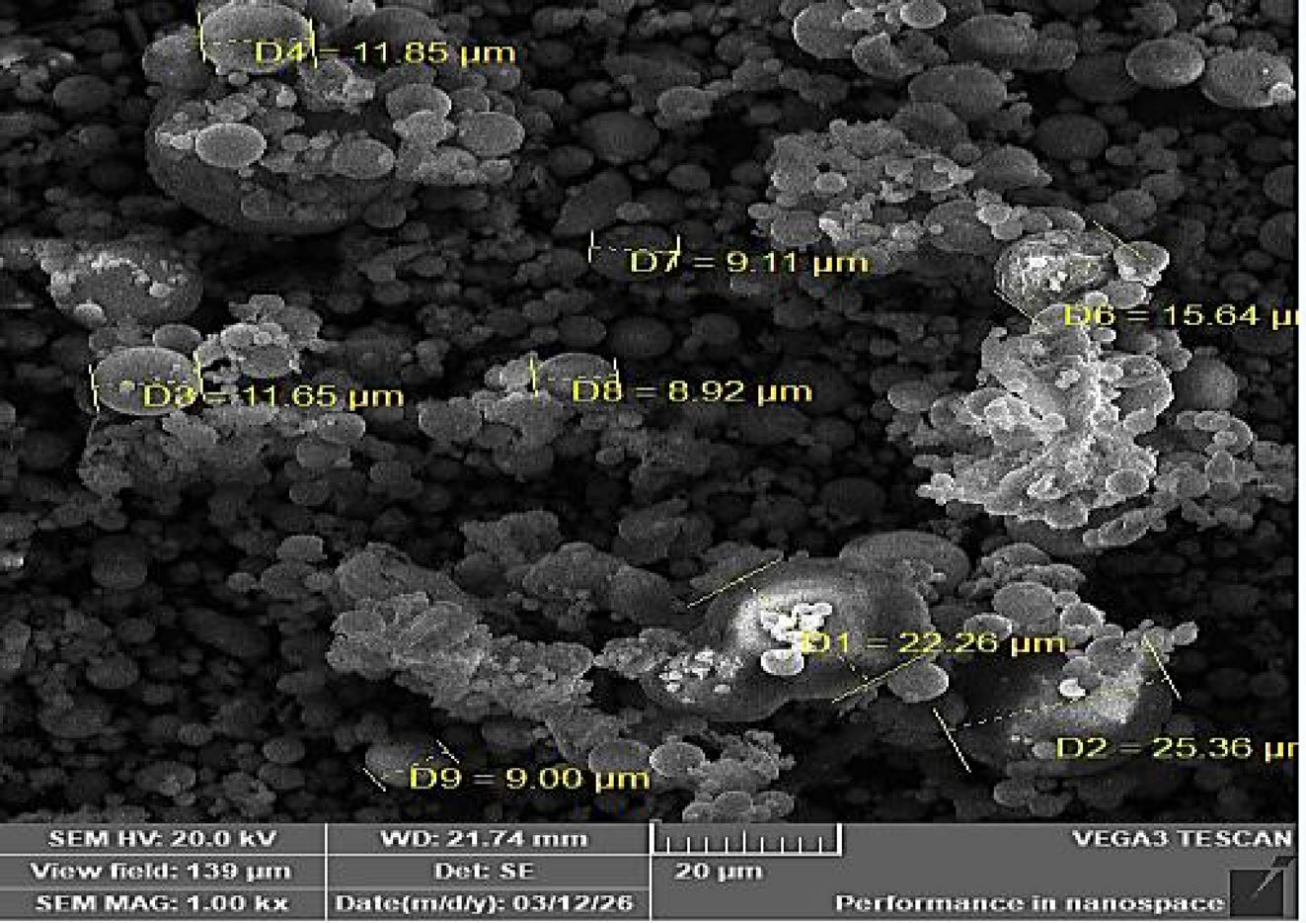
SEM micrograph showing the spherical morphology of the fly ash.

**Fig. 4:**
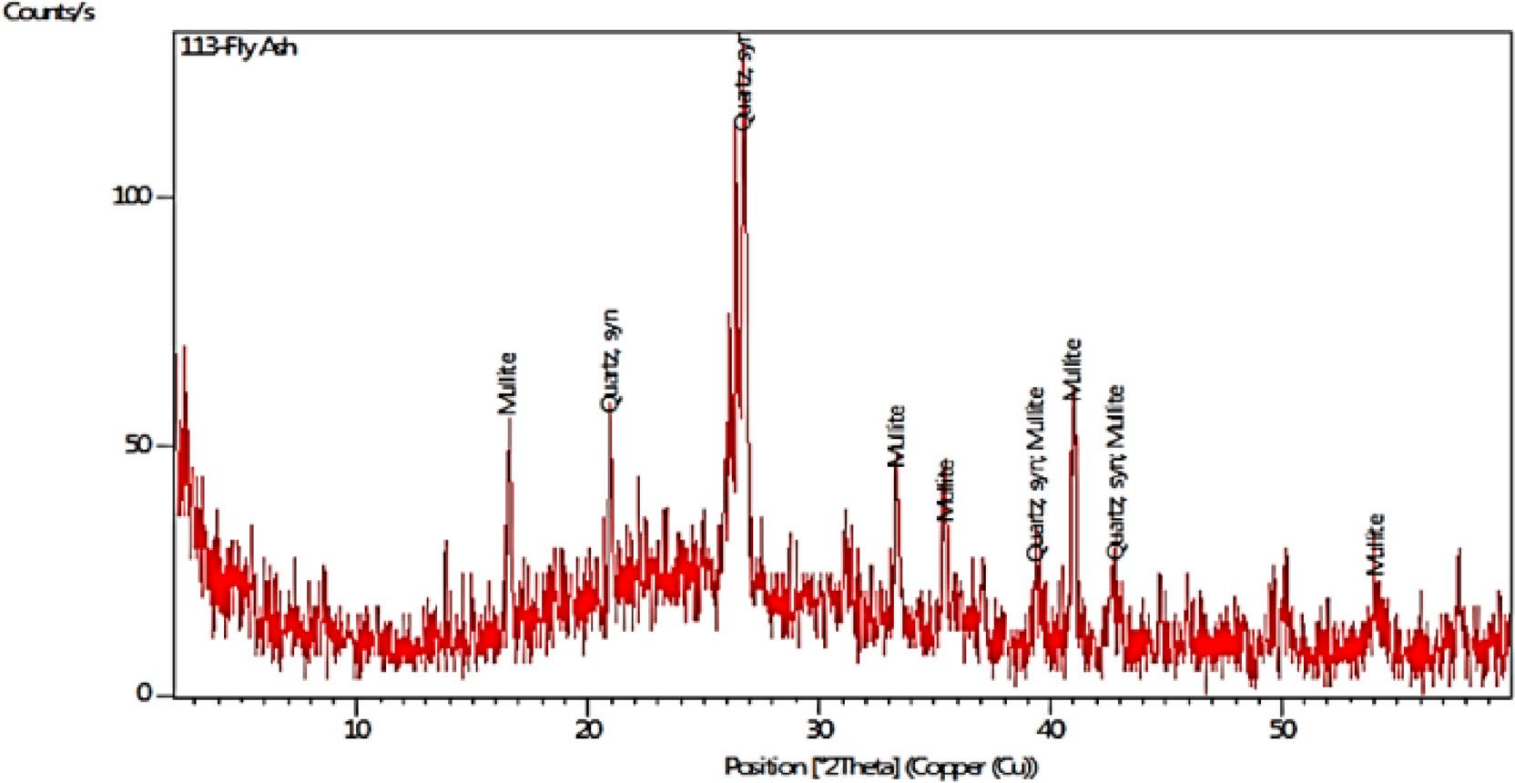
X-ray diffraction (XRD) pattern of fly ash exhibiting crystalline and amorphous phases.

Class F fly ash (FA) complying with ASTM C618-19 requirements^37^ served as the primary aluminosilicate precursor for the geopolymer concrete. The FA exhibited a characteristic grey appearance (Fig. 2) and was stored under dry conditions to mitigate moisture-induced degradation. Morphological evaluation via SEM analysis revealed predominantly smooth, spherical glassy particles with diameters ranging from 9 μm to 25 μm (Fig. 3). This spherical morphology enhances the workability of the fresh mixture via the ball-bearing effect, while concurrently facilitating gradual pozzolanic reactivity. Furthermore, XRD analysis identified quartz and mullite as the dominant crystalline phases, accompanied by a broad amorphous hump that signifies a high content of reactive vitreous phases (Fig. 4). These mineralogical findings correlate well with the chemical composition of the FA (Tables 2 and 3), which is characterized by a low CaO content and a high concentration of SiO_2_ and Al_2_O_3_.

**Table 2:** Physical characteristics of fly ash.

Parameter	Value
Specific Surface area (Blaine)	320 m²/kg
Fineness on 45 μm sieve	15%
Colour	Grey
Specific gravity	2.1
Bulk density	1000 kg/m^3^

**Table 3:** Chemical Composition (wt%) of Fly ash.

Parameter	SiO₂	Al₂O₃	Fe₂O₃	CaO	MgO	SO₃	Na₂O	Cl	L.O.I
Percentage %	52	37	1	5	4	3	1	0.05	2
ASTM C618-19 [39]	SiO₂+ Al₂O₃+ Fe₂O₃≥70%			≤ 18%					≤ 6

The Reactive SiO₂ content of the fly ash was determined to be 32.00 wt% of the total mass.

#### Sugarcane bagasse ash

Sugarcane bagasse ash (SCBA) represents a silica-rich agricultural by-product that serves as an alternative aluminosilicate precursor in geopolymer concrete. Under alkaline activation, it participates in the formation of Si–O–Al bonds, contributing to the development of a dense geopolymeric matrix.

**Fig. 5:**
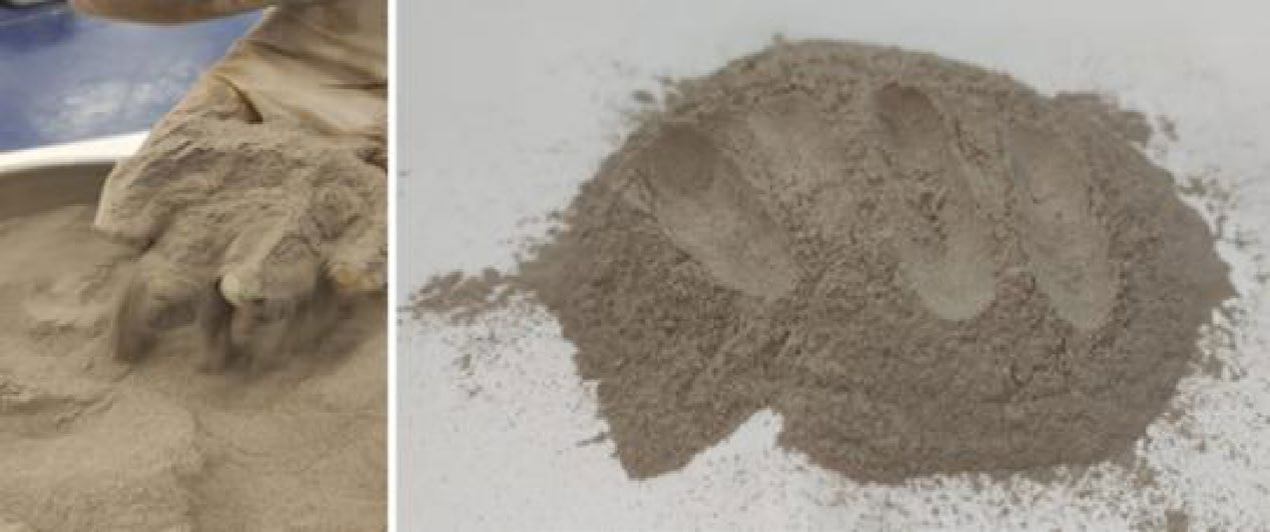
Visual appearance of the sugarcane bagasse ash (SCBA) powder.

In this study, raw SCBA was obtained from the Sugar and Integrated Industries Company (SIIC) in Nag Hammadi, Egypt, where it was produced through industrial combustion of sugarcane bagasse in industrial boilers. To optimize particle size uniformity and enhance its pozzolanic reactivity, the raw ash was subsequently processed through 75 μm sieve, as illustrated in Fig. 5. The physical and chemical properties of the processed SCBA are presented in Tables 4 and 5, respectively, confirming its suitability and pozzolanic potential for geopolymer applications.

**Fig. 6:**
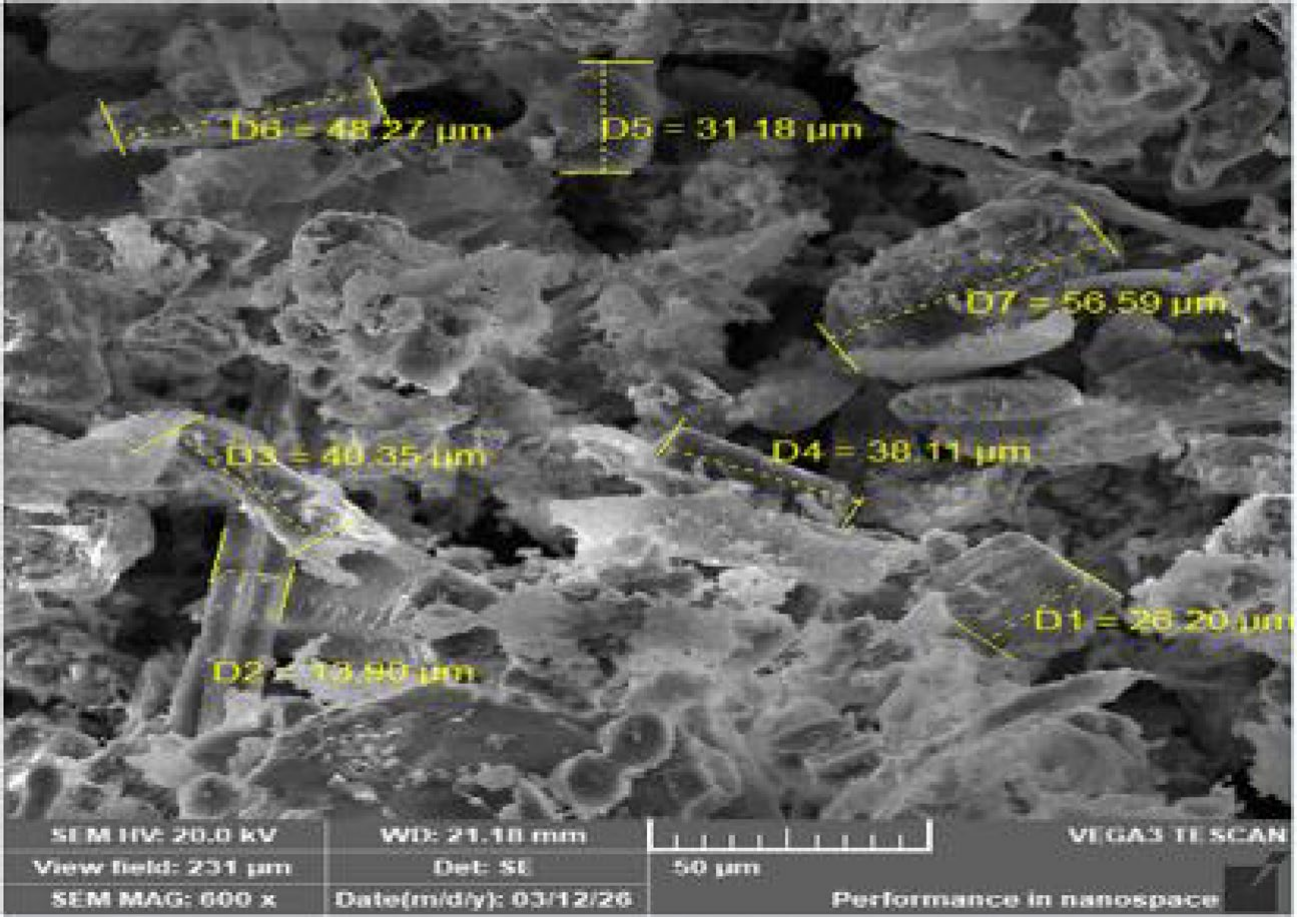
SEM micrograph highlighting the irregular, highly angular, and porous particle microstructure of the processed SCBA.

**Fig. 7:**
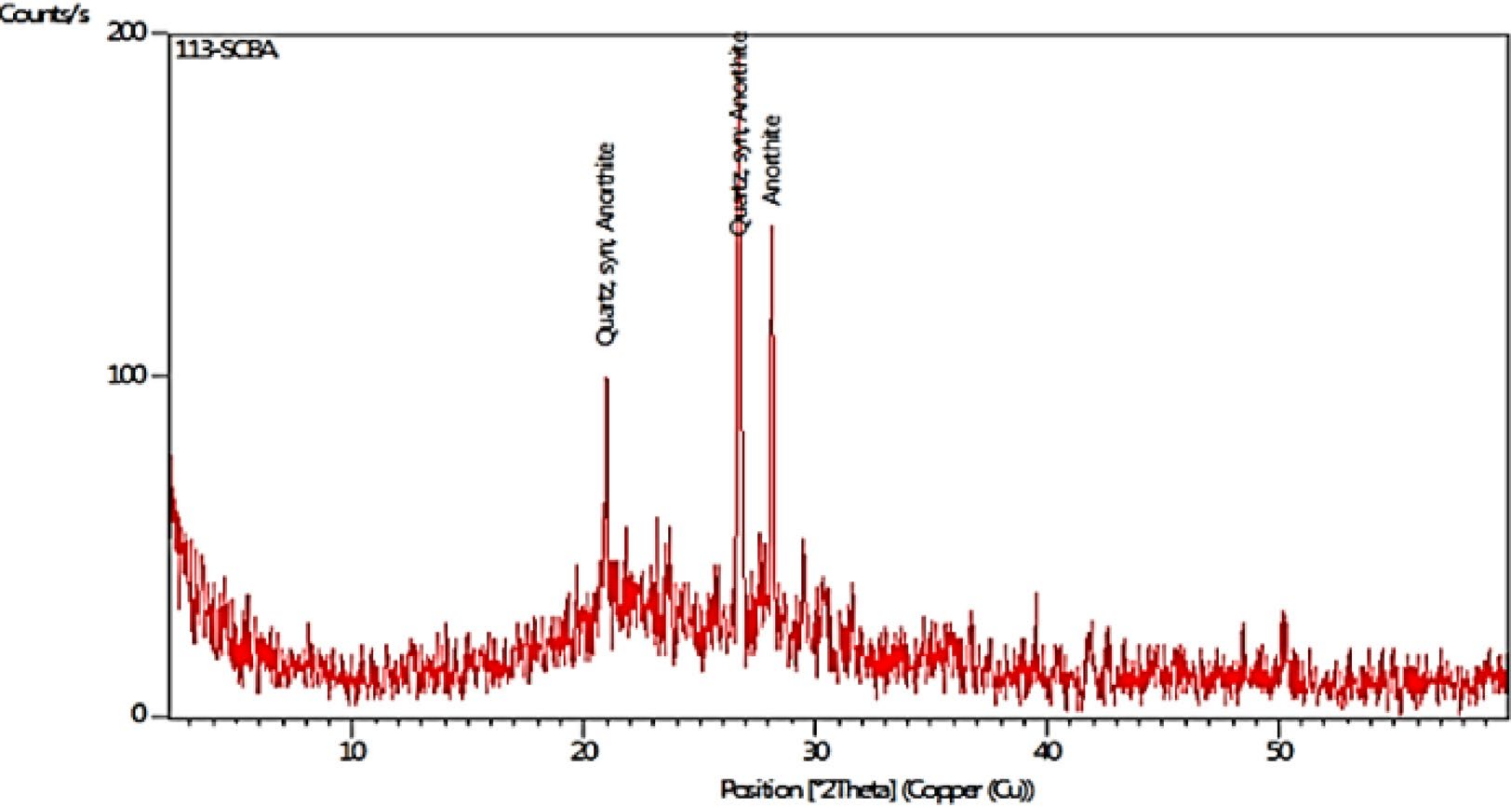
X-ray diffraction (XRD) spectrum of the processed SCBA showing intense crystalline quartz peaks.

Morphological evaluation via SEM analysis revealed that the SCBA consists of irregular, angular, and porous particles with sizes ranging from 13 μm to 56 μm (Fig. 6). This highly angular morphology enhances mechanical interlocking within the hardened matrix, but concurrently increases the liquid demand and reduces packing efficiency in the fresh state. Furthermore, XRD analysis indicated that the SCBA is predominantly composed of crystalline quartz, with minor anorthite phases and limited amorphous content, suggesting a relatively low intrinsic reactivity. These mineralogical findings are consistent with its chemical composition, which is characterized by a high SiO₂ content and relatively low Al₂O₃ content. Overall, SCBA behaves as a silica-rich material with limited intrinsic reactivity that contributes to the matrix mainly through filler effects and supplementary pozzolanic reactions. The XRD pattern of the raw SCBA is presented in Fig. 7.

**Table 4:** The physical characteristics of SCBA.

Parameter	Value
Colour	Grey
Bulk density	1138 kg/m^3^
Specific gravity	2.246
Average particle size	< 75 μm

**Table 5:** Chemical composition (wt%) of the processed SCBA.

Parameter	SiO_2_	AL_2_O_3_	Fe_2_O_3_	CaO	Na_2_O	K_2_O	MgO	SO_3_	L.O.I
Percentage %	50.8	4.5	0.4	4.91	0.9	4.1	5.03	0.5	28.0

The relatively high LOI value of 28% indicates a substantial volatile fraction, which may include residual unburned carbon associated with incomplete combustion. It should be noted that LOI does not directly quantify carbon content. The carbonaceous and porous fraction of SCBA may increase the adsorption of the alkaline activator and superplasticizer, thereby increasing liquid demand and reducing fresh-mixture workability. It may also decrease the effective reactive fraction and influence precursor dissolution and geopolymeric gel formation, particularly at high SCBA contents. These effects may contribute to the increased porosity and reduced mechanical performance observed at higher replacement levels. However, the carbon content and batch-to-batch variability were not independently measured in the present study and should be investigated in future work.

#### Silica fume

**Fig. 8:**
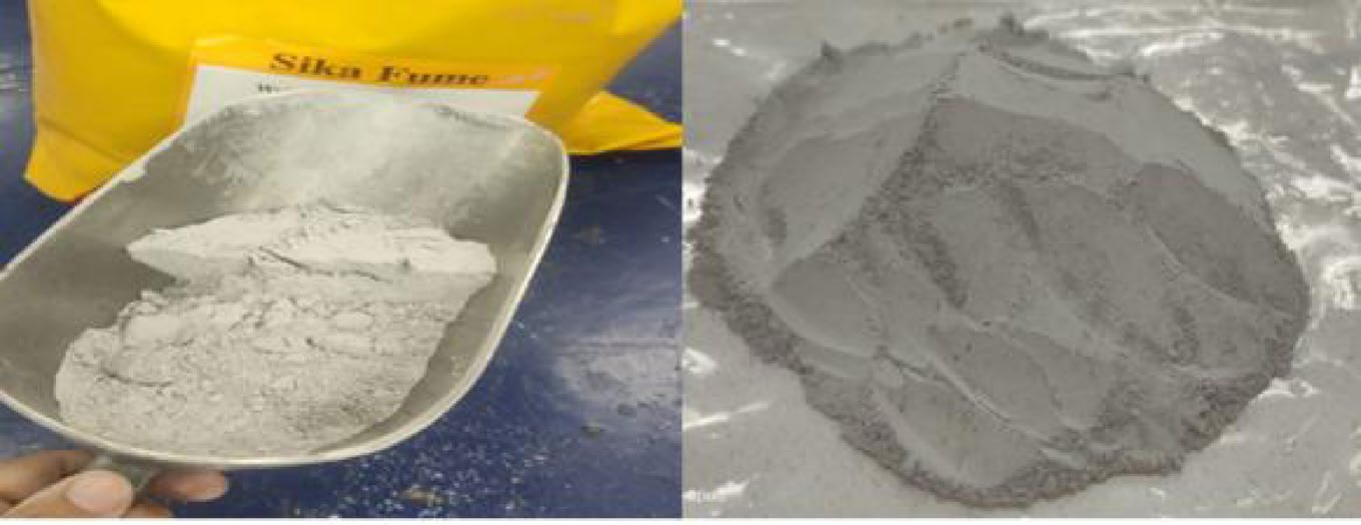
The visual appearance of the raw silica fume powder.

Silica fume constitutes an ultrafine by-product of ferrosilicon production, composed predominantly of highly reactive SiO₂ with a primary particle size of approximately 0.1 μm. It enhances geopolymer reactivity by filling micro-pores and promoting the development of a denser matrix. In this study, silica fume was incorporated as a supplementary pozzolanic material to improve the microstructure and matrix cohesion at a fixed dosage of 5% by weight of the total binder materials. Its physical properties are presented in Table 6, while Fig. 8 shows the visual appearance of the raw material.

**Fig. 9:**
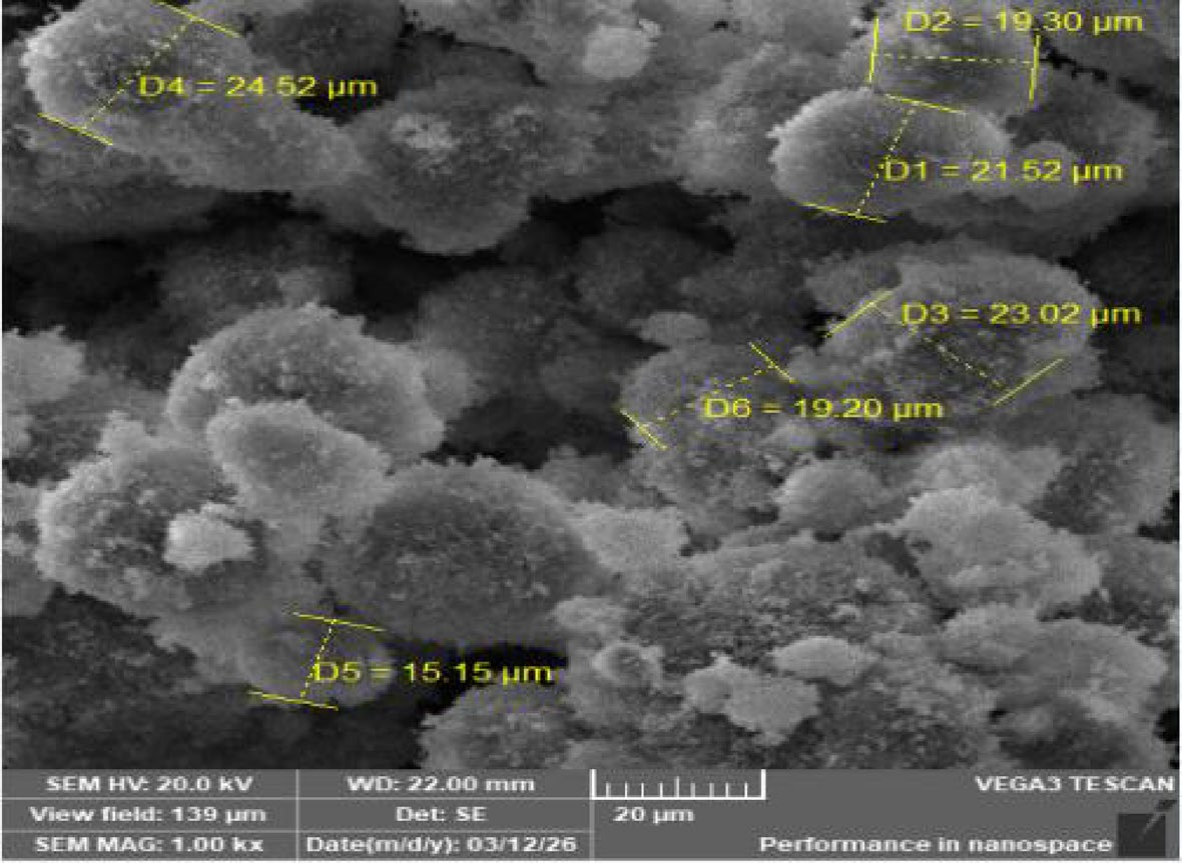
SEM micrograph showing the agglomerated clusters of ultrafine spherical silica fume particles.

**Fig. 10:**
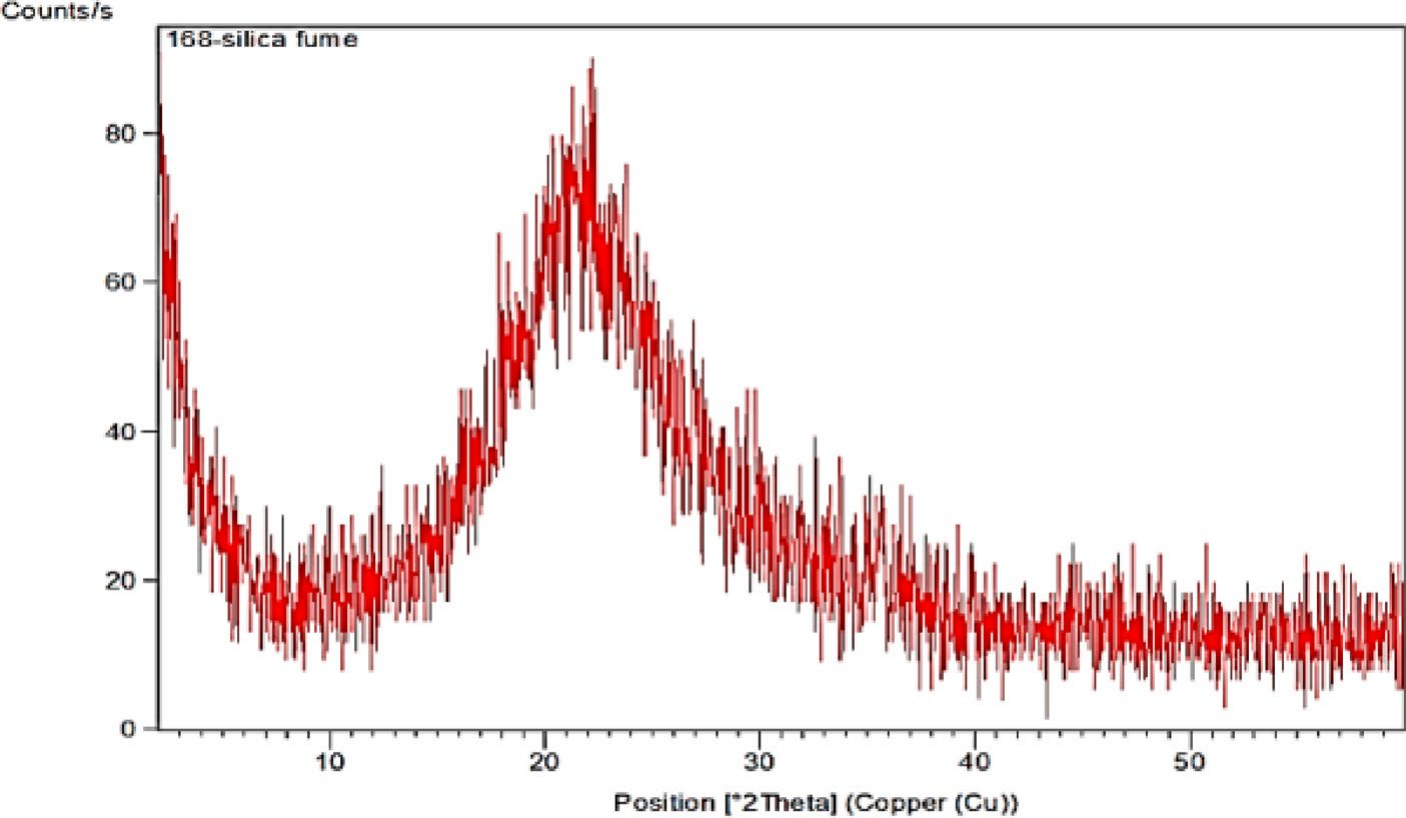
XRD pattern of the silica fume demonstrating its predominantly amorphous, glassy nature.

Morphological evaluation via SEM analysis revealed agglomerated clusters of ultrafine spherical particles with apparent sizes ranging from 15 μm to 25 μm (Fig. 9). These agglomerates reflect the extremely high specific surface area of silica fume, which enhances pozzolanic reactivity and improves void-filling capabilities, resulting in a denser microstructure and reduced porosity. Furthermore, XRD analysis exhibited a broad amorphous hump without distinct crystalline peaks, indicating the predominance of a highly reactive glassy phase. This observation is consistent with its chemical composition, which contains more than 90% SiO₂ and a negligible CaO content, confirming its highly siliceous and reactive nature. Owing to the absence of significant inert constituents, silica fume readily participates in the geopolymerization reactions and contributes directly to gel formation. The XRD pattern of the silica fume is presented in Fig. 10.

**Table 6:** The characteristics of silica fume.

Parameter	Value
Specific Surface area (Blaine)	> 1500 m²/kg
Colour	Grey
Bulk density	300 kg/m^3^
Specific gravity	2
Average particle size (µm)	0.1

#### The alkaline activator solutions

The alkaline activator system used in this study consisted of sodium hydroxide (NaOH) solutions with molarities of 12 M, 14 M, and 16 M, combined with sodium silicate (Na₂SiO₃) solution. To ensure consistency among the fifteen geopolymer concrete mixtures, the Na₂SiO₃/NaOH mass ratio, alkaline solution-to-binder mass ratio, and total binder content were kept constant at 2.5, 0.45, and 400 kg/m³, respectively. The measured densities of the prepared NaOH solutions were 1.35 g/cm³, 1.40 g/cm³, and 1.435 g/cm³ for the 12 M, 14 M, and 16 M concentrations, respectively.

To isolate the effect of NaOH molarity, the total mass of the NaOH solution incorporated into each geopolymer mixture was maintained constant at 51.43 kg/m³. The required NaOH solutions (12 M, 14 M, and 16 M) were prepared by dissolving different masses of NaOH flakes in corresponding amounts of distilled water while maintaining the same total solution mass. Consequently, the mass of NaOH flakes and the water content varied according to the target molarity, whereas the total NaOH solution mass added to each mixture remained constant. The Na₂SiO₃ solution mass was also kept constant, maintaining a fixed Na₂SiO₃/NaOH solution mass ratio of 2.5 throughout the experimental program.

**Fig. 11:**
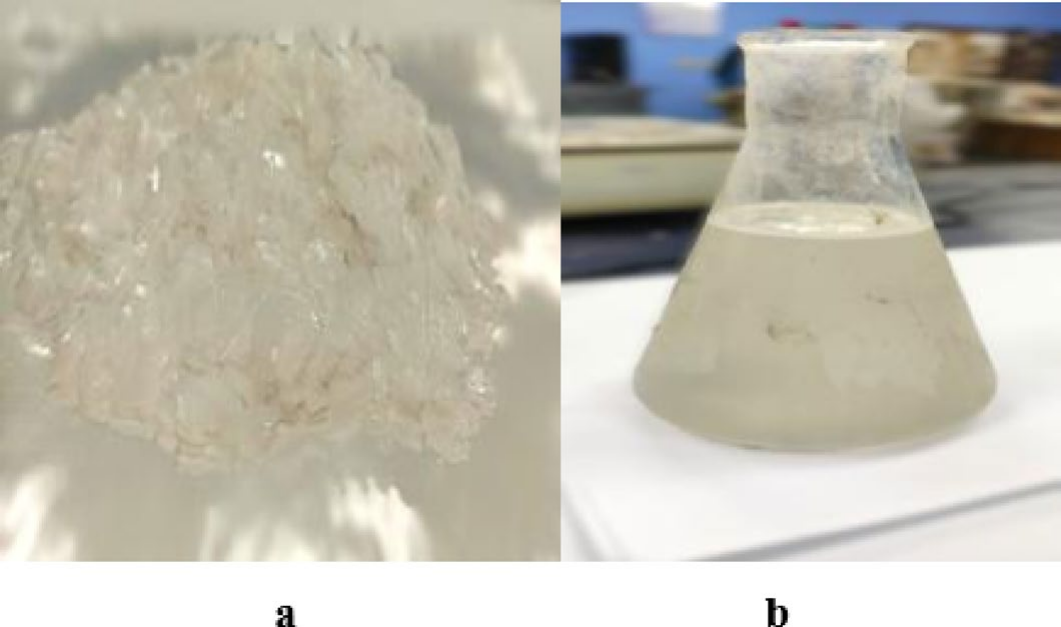
Visual appearance of (**a**) high-purity sodium hydroxide flakes prior to dissolution, and (**b**) the combined alkaline activator solutions after mixing and stabilization.

Functionally, the alkaline activator governs aluminosilicate dissolution and the subsequent formation of Si–O–Al geopolymeric gels. NaOH promotes the dissolution of aluminosilicate species from the precursor materials, whereas sodium silicate provides additional soluble silica and enhances polymeric network formation. The NaOH solutions of 12 M, 14 M, and 16 M were prepared by dissolving the calculated mass of high-purity NaOH flakes in distilled water according to Eq. (1). Different quantities of NaOH flakes and water were used for each molarity, as presented in Table 7 while maintaining a constant total NaOH solution mass. After cooling to room temperature, the NaOH solution was mixed with sodium silicate solution and stored for 24 h prior to casting to improve chemical stability and minimize concentration fluctuations. Figure 11 shows the visual appearance of the prepared alkaline activator solutions.

1 $$Mass{\text{ }}of{\text{ }}NaOH{\text{ }}\left( g \right){\text{ }} = {\text{ }}Molarity{\text{ }}\left( {mol/L} \right){\text{ }} \times {\text{ }}Volume{\text{ }}\left( L \right){\text{ }} \times {\text{ }}Molecular{\text{ }}Weight{\text{ }}\left( {g/mol} \right)$$

**Table 7:** Preparation of NaOH solutions with different molarities while maintaining a constant total solution mass.

Total NaOH solution (kg/m³)	Total NaOH solution density (Kg/L)	Total NaOH solution (Liter/m³)	Water (kg/m³)	NaOH flakes (kg/m³)	NaOH Molarity
51.43	1.35	38.096	33.144	18.286	12 M
51.43	1.4	36.736	30.858	20.572	14 M
51.43	1.435	35.840	28.493	22.937	16 M

#### Superplasticizer

**Fig. 12:**
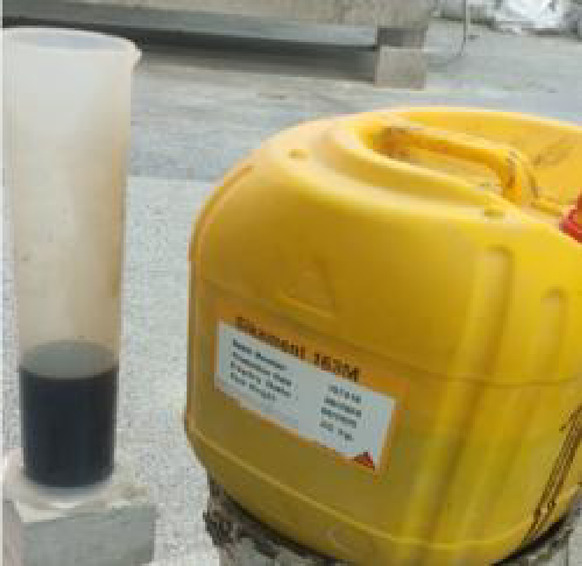
The superplasticizer utilized.

A high-range water-reducing admixture (Sikament-163 M), as illustrated in Fig. 12, was incorporated to improve the workability and compensate for the high viscosity inherent to the alkaline activator solution. The admixture exhibits a density of 1.2 g/cm³, was used at a fixed dosage of 2.5% by weight of the total binder, the utilized superplasticizer strictly complies with ASTM C494 Type F requirements^36^.

#### Aggregates

Natural siliceous sand and crushed dolomite with a nominal maximum size (NMS) of 10 mm were utilized as the fine and coarse aggregates, respectively. The physical properties of the materials were experimentally determined; the sand exhibited a bulk density of 1714 kg/m³ and a specific gravity of 2.58, whereas the crushed dolomite displayed a bulk density of 1687 kg/m³ and a specific gravity of 2.69.

The particle size distribution of each aggregate was verified via sieve analysis, confirming strict compliance with the Egyptian Code of Practice (ECP 203) requirements^38^. The fine aggregate possessed a fineness modulus of 2.35, indicating a well-graded fine-to-medium sand. According to the (ECP 203), the grading curve of the utilized sand falls precisely within Zone 2, which represents the ideal grading envelope for producing structural concrete mixtures.

To optimize particle packing efficiency and minimize interstitial void volume within the geopolymeric skeleton, a combined aggregate matrix was engineered by blending the natural sand and crushed dolomite at a controlled weight ratio of 1:1.600, corresponding to 38.48% fine and 61.52% coarse aggregates. The comprehensive particle size distribution of the individual components, along with the computed continuous grading of the blended matrix, is detailed horizontally in Table 8.

**Fig. 13:**
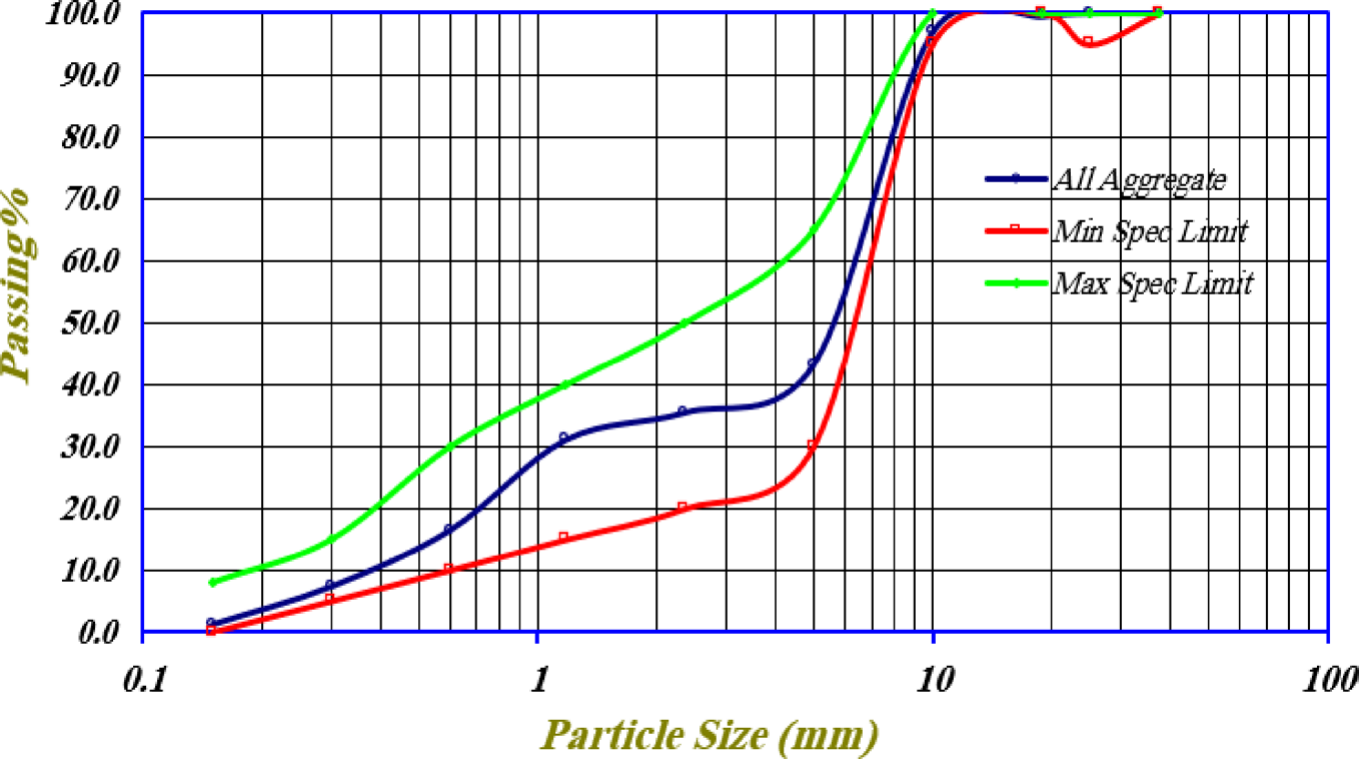
Sieve analysis of combined aggregate compared with ECP 203 limits.

As illustrated by the combined grading curves in Fig. 13, the optimized aggregate blend (the Combined Mix) follows a seamless S-shaped profile that lies entirely within the upper and lower specification limits. The distinctive gradation drop between the 10 mm (96.9% passing) and 5 mm (43.3% passing) sieves indicates an excellent interlocking structure that prevents segregation while concurrently ensuring sufficient fresh workability and rheological stability during casting.

**Table 8:** Sieve analysis of individual aggregates and the resulting combined aggregate matrix compared with standard specification limits.

Sieve (mm)	37.5	25	20	10	5	2.36	1.18	0.6	0.3	0.15
Fine aggregate	100	100	100	100	100	91.6	80.9	43	19.2	3.3
Coarse aggregate	100	100	99.2	95	7.8	0.4	0	0	0	0
Lower limit	-	-	100	95	30	20	15	10	5	0
Upper limit	-	-	100	100	65	50	40	30	15	8

### Specimens preparation

The geopolymer concrete mixtures were fabricated utilizing a mechanical pan mixer with a specific mixing sequence to ensure matrix homogeneity. Initially, the solid constituents—including the precursor materials (fly ash and sugarcane bagasse ash) and the fine and coarse aggregates—were dry-mixed for 3 min. Concurrently, the pre-prepared alkaline activator solution and the high-range water-reducing admixture were thoroughly blended and then gradually introduced into the dry matrix. The entire batch was subsequently wet-mixed for an additional 4 min until a uniform and cohesive fresh consistency was achieved.

**Fig. 14:**
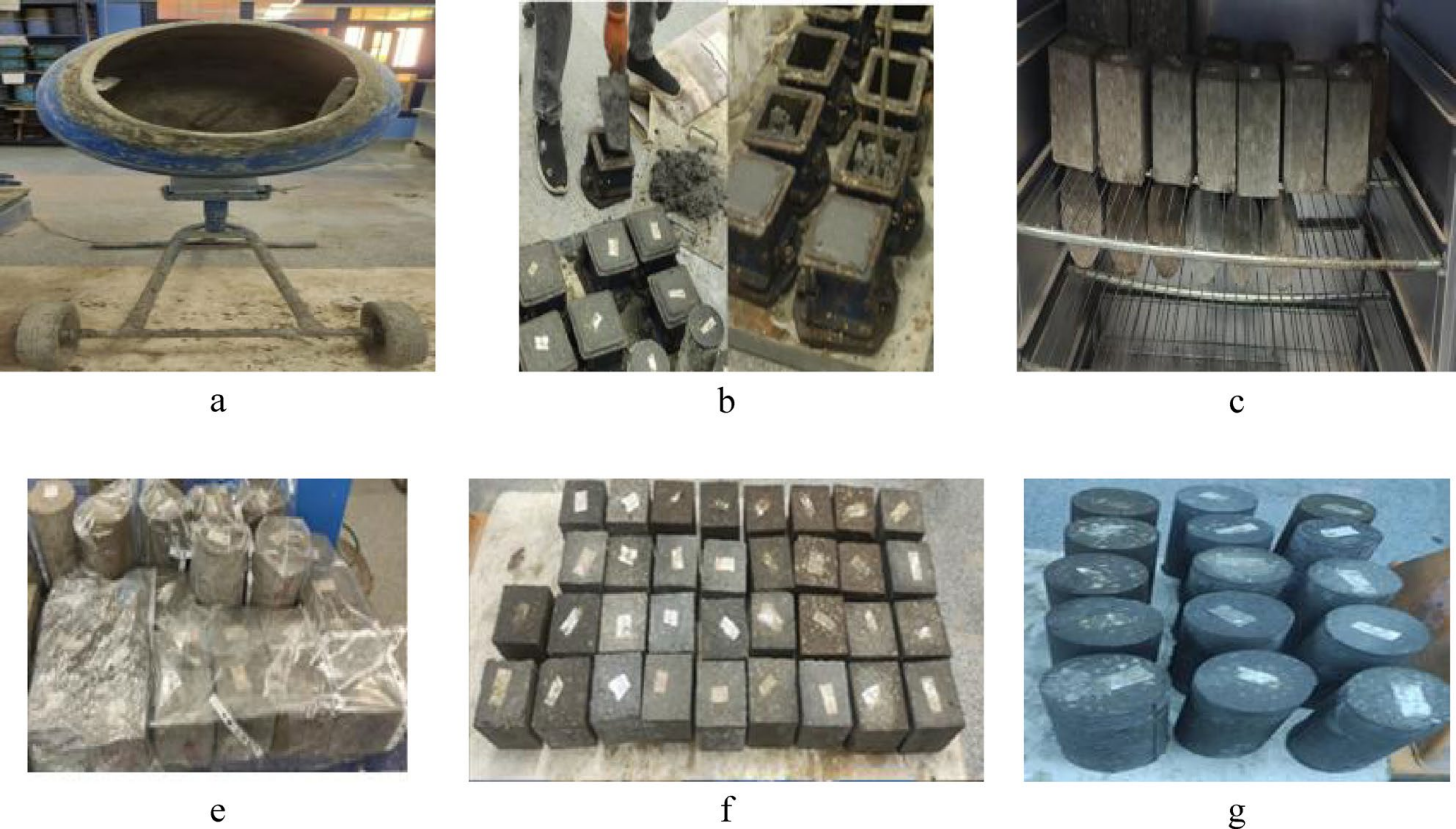
Sequential operational stages of geopolymer concrete preparation; (**a**) mixing, (**b**) molding, (**c**,**d**) curing, and (**f**,**g**) cubes and cylinders.

For mechanical performance and durability characterization, cubic specimens (100 mm) and cylindrical specimens (100 × 200 mm) were cast and compacted in accordance with ASTM C192/C192M guidelines^39^. Following the casting operation, the specimens were securely kept in the laboratory environment. After 24 h, the specimens were carefully demolded and subjected to thermal curing in a calibrated oven at 70 °C for a duration of 24 h. Upon completion of the heat-curing period, the specimens were allowed to cool gradually to room temperature and remained sealed under plastic wrapping to maintain a stable internal relative humidity until the designated testing ages. The sequential operational stages of the geopolymer concrete preparation are schematically illustrated in Fig. 14.

## Experimental testing program

### The slump tests

**Fig. 15:**
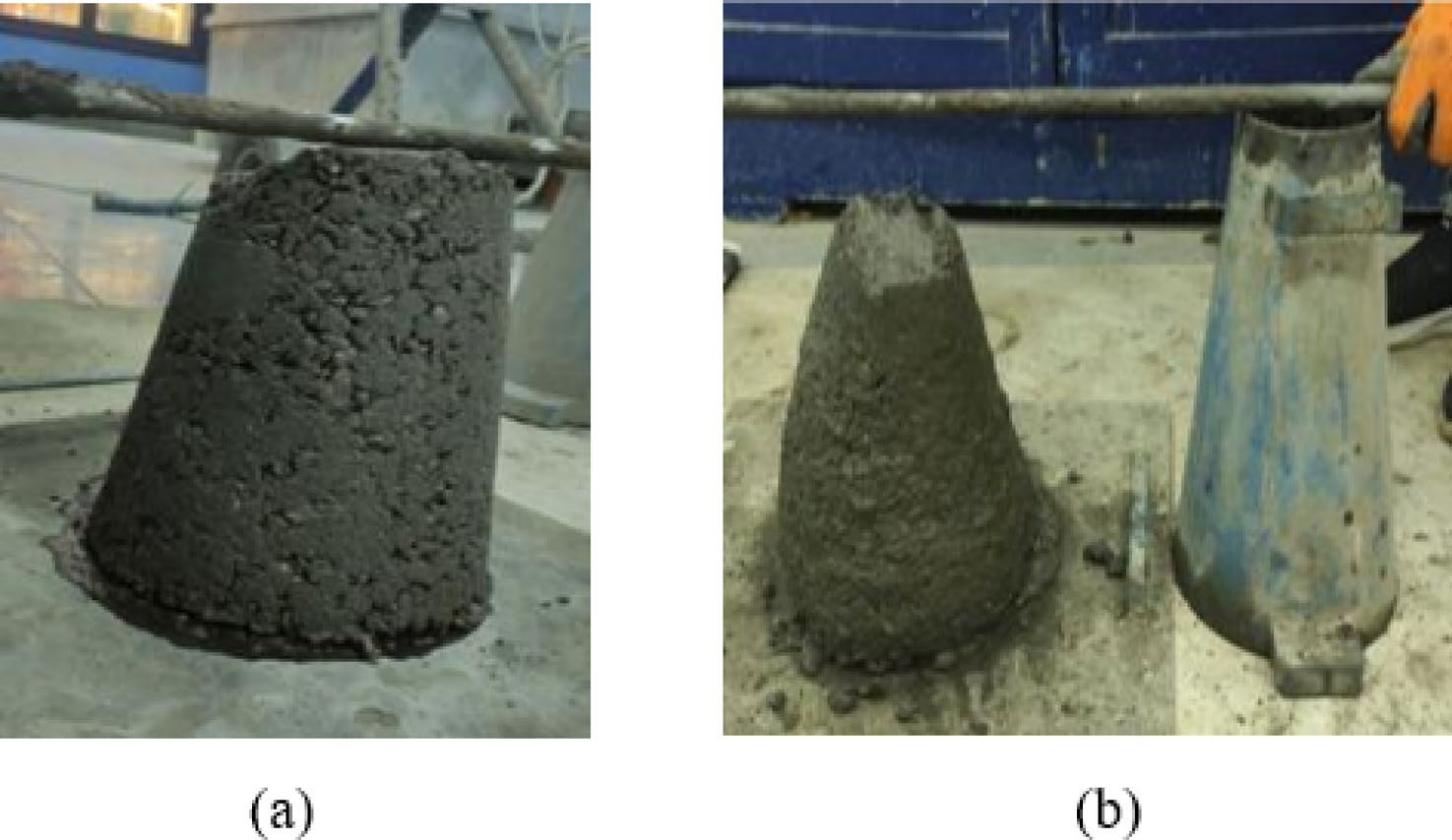
Effect of superplasticizer addition on the workability of geopolymer concrete: (**a**) without superplasticizer and (**b**) with superplasticizer.

The slump test was conducted in accordance with ASTM C143^40^ to evaluate the workability of the geopolymer concrete mixtures. The slump cone was filled in three equal layers by volume, with each layer compacted with 25 strokes of a tamping rod before placing the next. After lifting the cone vertically, the slump value was measured. To assess the effectiveness of the superplasticizer, a preliminary comparison was performed between mixtures with and without its addition. As shown in Fig. 15, the incorporation of 2.5% superplasticizer by weight of the binder significantly improved workability and facilitated proper mixing and casting. Accordingly, the same dosage was adopted for all mixtures throughout the experimental program.

### Density

Density was determined for cube specimens in accordance with ASTM C642^41^, by measuring mass and dividing by volume, according to Eq. (2).

2 $$\:\rho\:=\frac{m}{v}$$

Where $\:{\uprho\:}$ is density, m is mass, and v is volume.

### Compressive strength

Compressive strength was determined using three cubes (100 × 100 × 100 mm) at 7, 28, 60, and 90 days in accordance with BS EN 12390-3^42^. Load was applied at a constant rate until failure. Strength was calculated Eq. (3).

3 $$\:fc=\frac{P}{A}$$

**Fig. 16:**
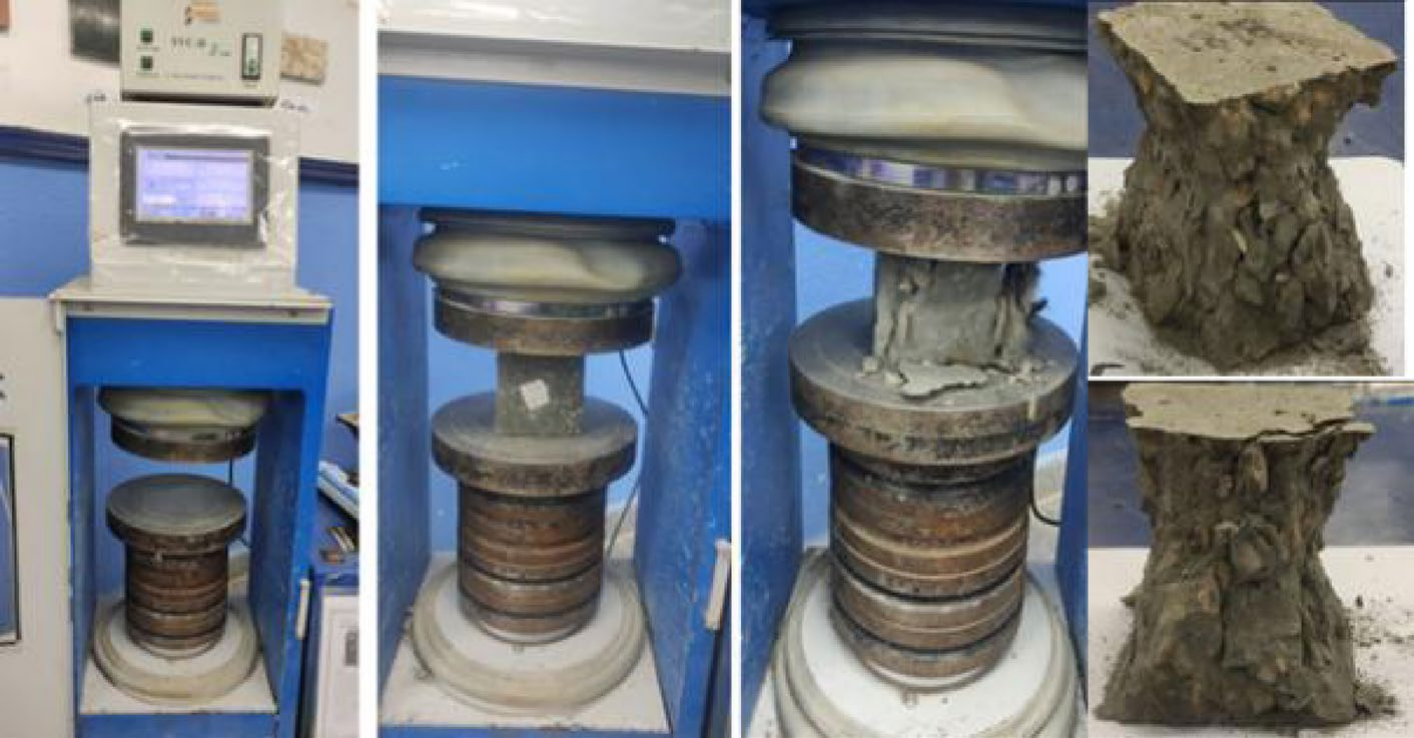
The compressive strength test.

Where P is the failure load and A is the cross-sectional area. Reported values represent the average of three specimens. Figure 16 illustrate the compression test.

### Indirect split tensile strength test

Splitting tensile strength was measured using three cylinders (100 × 200 mm) at 7, 28, 60, and 90 days in accordance with ASTM C496^43^. Specimens were loaded diametrically at a constant rate until failure. Strength was calculated as Eq. (4).

4 $$\:ft=\frac{2P}{\pi\:DL}$$

**Fig. 17:**
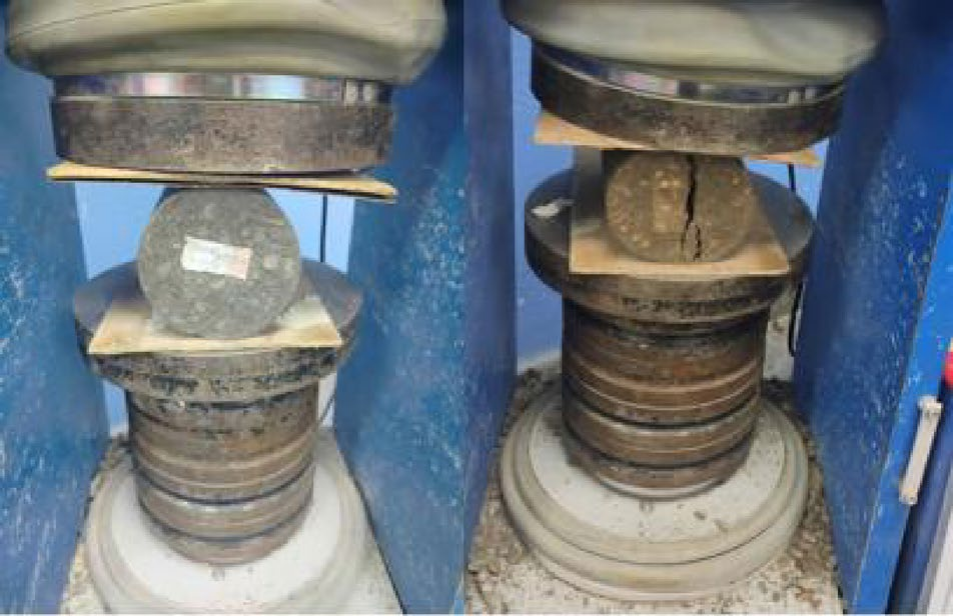
The Indirect split tensile test.

Where P is the failure load, L is length, and D is diameter. Results represent the average of three specimens. Figure 17 shows the indirect split tensile test setup.

### Schmidt hammer test

**Fig. 18:**
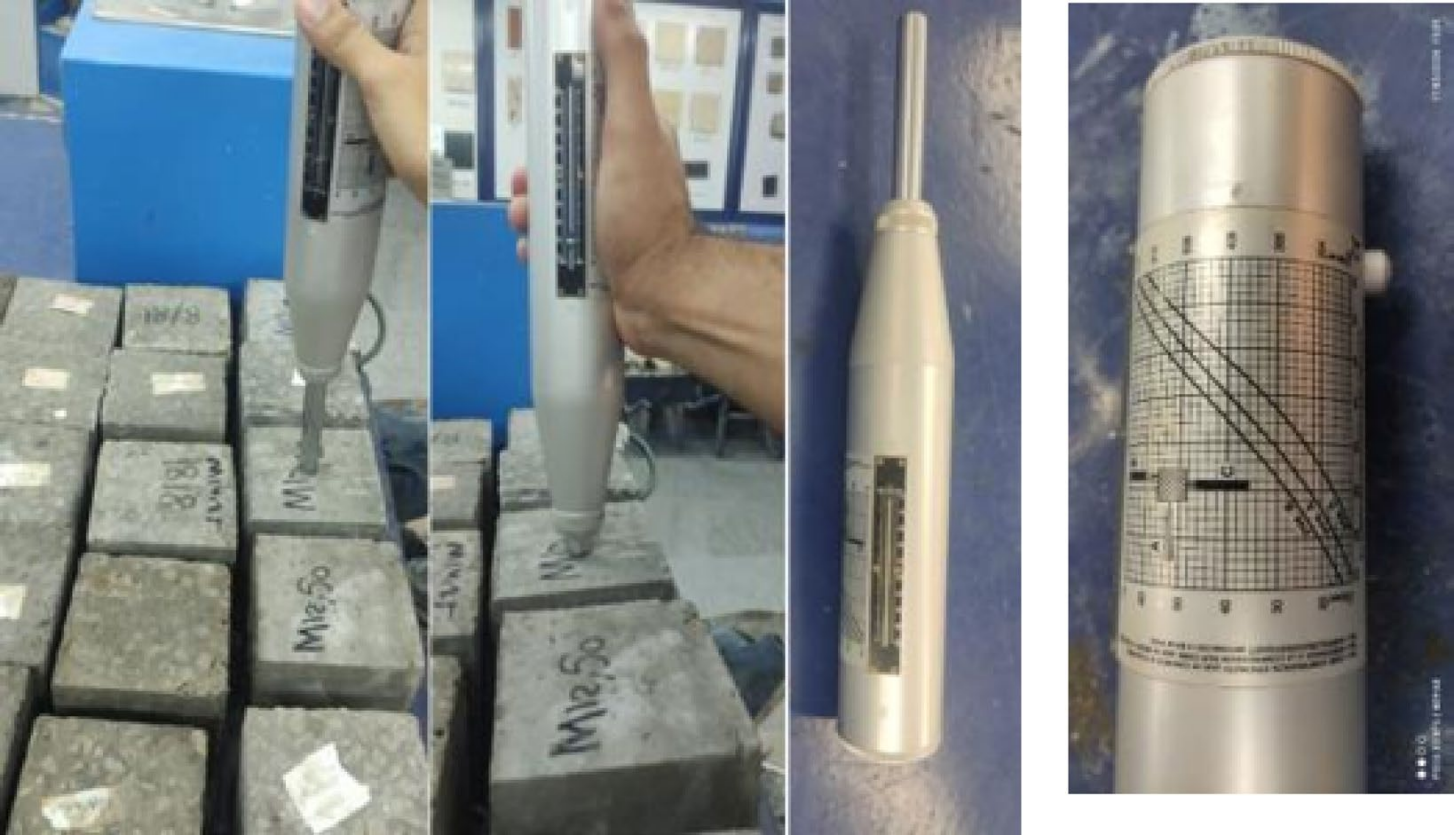
The Schmidt hummer test.

Schmidt hammer test was conducted at 28 days on 100 mm cubes to assess surface hardness in accordance with ASTM C805^44^. At least 10 readings were taken at different points away from edges. After excluding outliers, the average rebound number was calculated, and the corresponding compressive strength was estimated using the calibration curve. Figure 18 shows the test setup.

### Sorptivity test

**Fig. 19:**
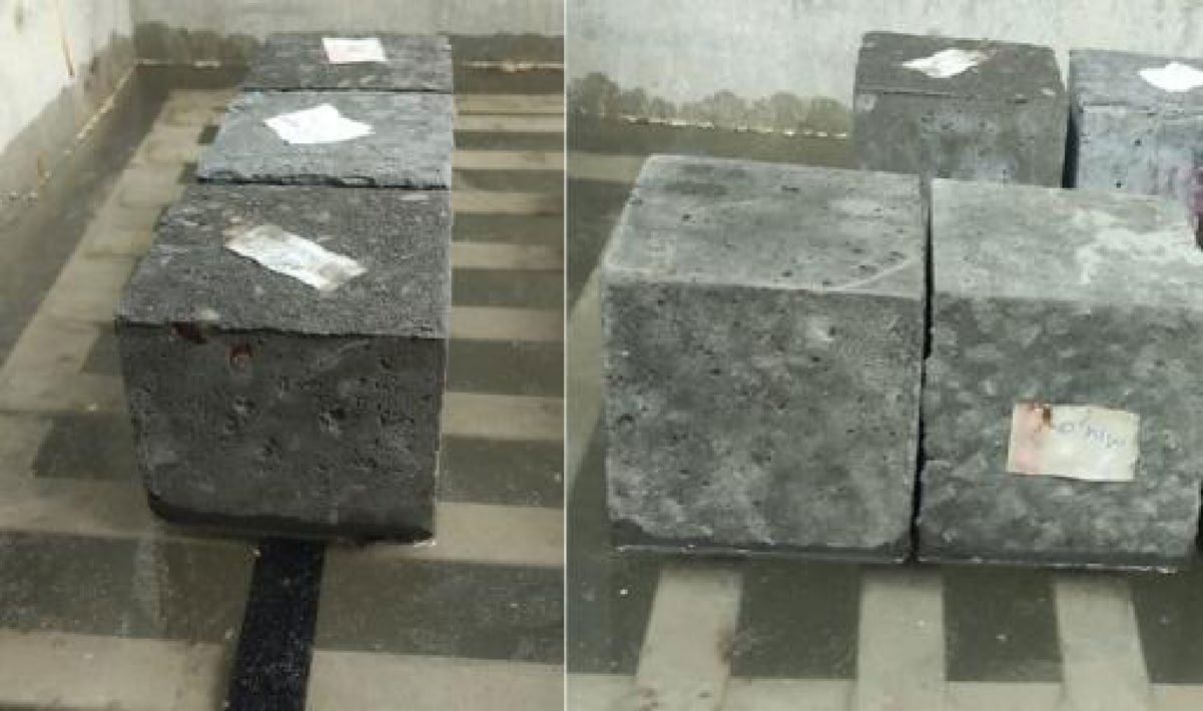
The sorptivity test.

Sorptivity was measured according to ASTM C1585/C1585M^45^ to evaluate the capillary water absorption rate of the geopolymer concrete. For each mixture, a minimum of three replicate specimens were tested to ensure statistical consistency. Cube specimens were dried, and exposed to water, as illustrated in Fig. 19, with their mass recorded over time. The cumulative water absorption (I) was calculated using Eq. (5). The initial and secondary sorptivity coefficients were compiled from the slope of $\:\frac{I}{\surd\:t}$, as expressed by Eq. (6):

5 $$\:I=\frac{mt-m0}{A\times\:\rho\:}$$

6 $$\:S=\frac{I}{\surd\:t}$$

Where mt−m0 is the increase in specimen mass (g), *A* is the area of the exposed surface (mm²), and *ρ* is the density of water (g/mm³), *I* is the cumulative water absorption (mm), *t* is time (s), and *S* is the sorptivity coefficient (mm/√s).

### Performance of geopolymer concrete at elevated temperatures

**Fig. 20:**
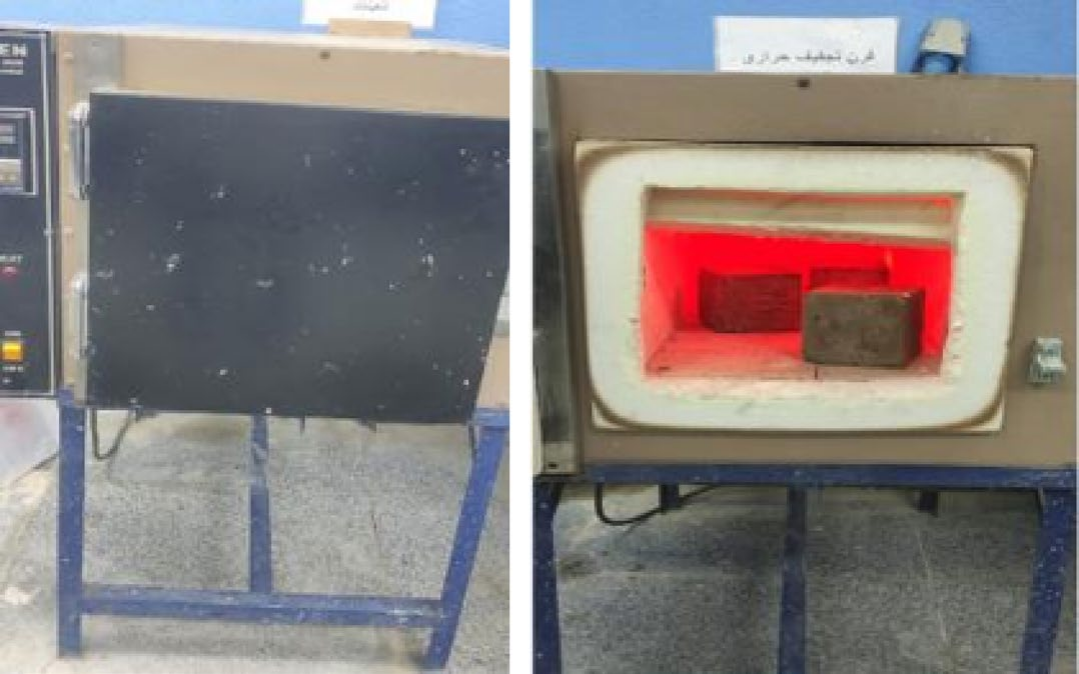
The electric laboratory furnace utilized for conducting elevated temperature tests.

Thermal behavior was assessed by heating cube and cylindrical specimens to 100–500 °C for two hours at a rate of 5 °C/min. To reduce thermal gradients, specimens were cooled in the furnace to room temperature. Residual compressive and tensile strengths were determined, along with mass loss and visual assessment of surface damage Mass loss was computed as Eq. (7). Figure 20 displays the furnace used.

7 $$\:Mass\:loss\:\%=\frac{W0-WT}{W0}\times\:100$$

Where W0 is the initial dry mass and WT is the final mass after heat exposure.

### Microstructure characteristics

To investigate the matrix morphology, elemental composition, phase assemblage, and geopolymerization products, microstructural characterization was performed using scanning electron microscopy (SEM) coupled with energy-dispersive X-ray spectroscopy (EDX) and X-ray diffraction (XRD). All analyses were conducted at the National Research Centre, Giza, Egypt. A Tescan VEGA3 scanning electron microscope was used for the SEM/EDX investigation. The morphology and elemental composition of dried, gold-coated fragments obtained from crushed concrete specimens were systematically examined. SEM/EDX and XRD analyses were performed on selected representative mixtures rather than all fifteen investigated mixtures. The selected specimens were chosen to represent the principal variables of the experimental program, including the control mixture, the B25 mixture, higher SCBA contents, different NaOH molarities, and selected elevated-temperature exposure conditions., different NaOH molarities, and selected elevated-temperature exposure conditions. This representative selection was intended to identify the microstructural changes associated with the investigated variables and to provide representative evidence supporting the trends observed in the mechanical, durability, and thermal performance results. Because only selected specimens were examined, the microstructural observations should be interpreted as representative evidence and should not be generalized to all fifteen mixtures without further characterization.

## Results and discussion

### Results of slump test

**Fig. 21:**
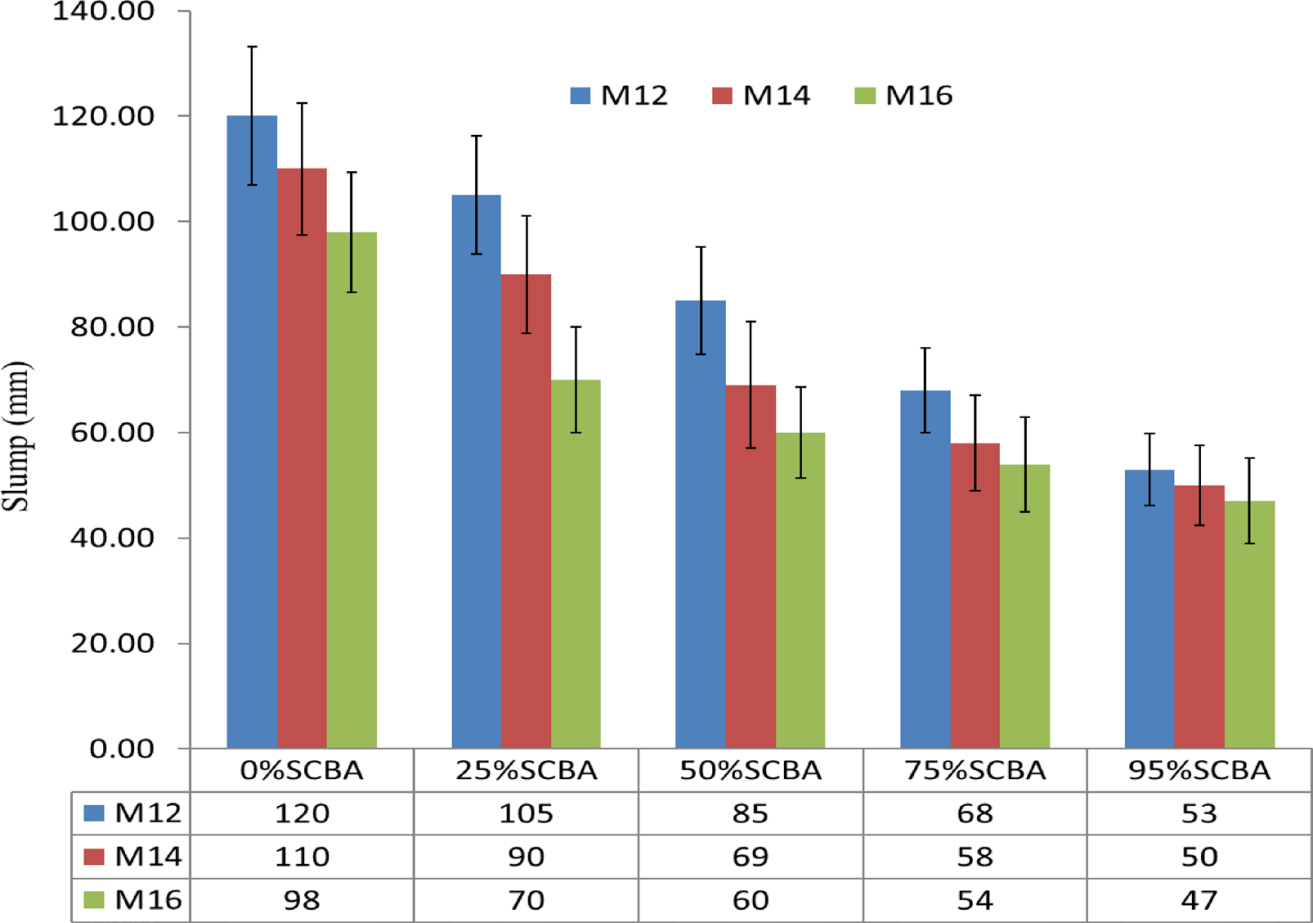
Slump test results presented as mean values of three measurements (*n* = 3). Error bars represent standard deviation.

As illustrated in Fig. 21, the slump values progressively decreased from a maximum of 120 mm (at 0% SCBA with 12 M NaOH) to a minimum of 47 mm (at 95% SCBA with 16 M NaOH) as a function of increasing sugarcane bagasse ash (SCBA) content and NaOH molarity. Notably, the B25 mixture (containing 25% SCBA and prepared with 14 M NaOH) exhibited a slump of 90 mm, demonstrating satisfactory and promising workability for practical applications. In comparing the different incorporation levels, the control mixtures (0% SCBA) maintained the highest fluidity across all molarities (98–120 mm). Conversely, a sharp decline in flowability was observed at higher replacement levels (75% and 95% SCBA), where slump values dropped below 70 mm and 55 mm, respectively, regardless of the NaOH concentration. This reduction in workability is primarily attributed to the high specific surface area and elevated liquid absorption capacity of the SCBA particles. The high LOI of the SCBA may have additionally contributed to this behavior through increased adsorption of the alkaline solution and superplasticizer by the residual carbonaceous fraction. Furthermore, the higher molarity of the alkaline solution increases its viscosity, which subsequently accelerates the geopolymerization kinetics and impedes mixture flowability. These findings align with the observations reported by N. Chuewangkam^35^. The standard deviation of the triplicate slump measurements ranged from 6.82 to 13.12 mm. Two-way ANOVA showed significant effects of SCBA content (F(4,30) = 49.10, *p* < 0.001) and NaOH molarity (F(2,30) = 15.38, *p* < 0.001), whereas their interaction was not statistically significant (F(8,30) = 0.96, *p* = 0.484).

### Results of density test

**Fig. 22:**
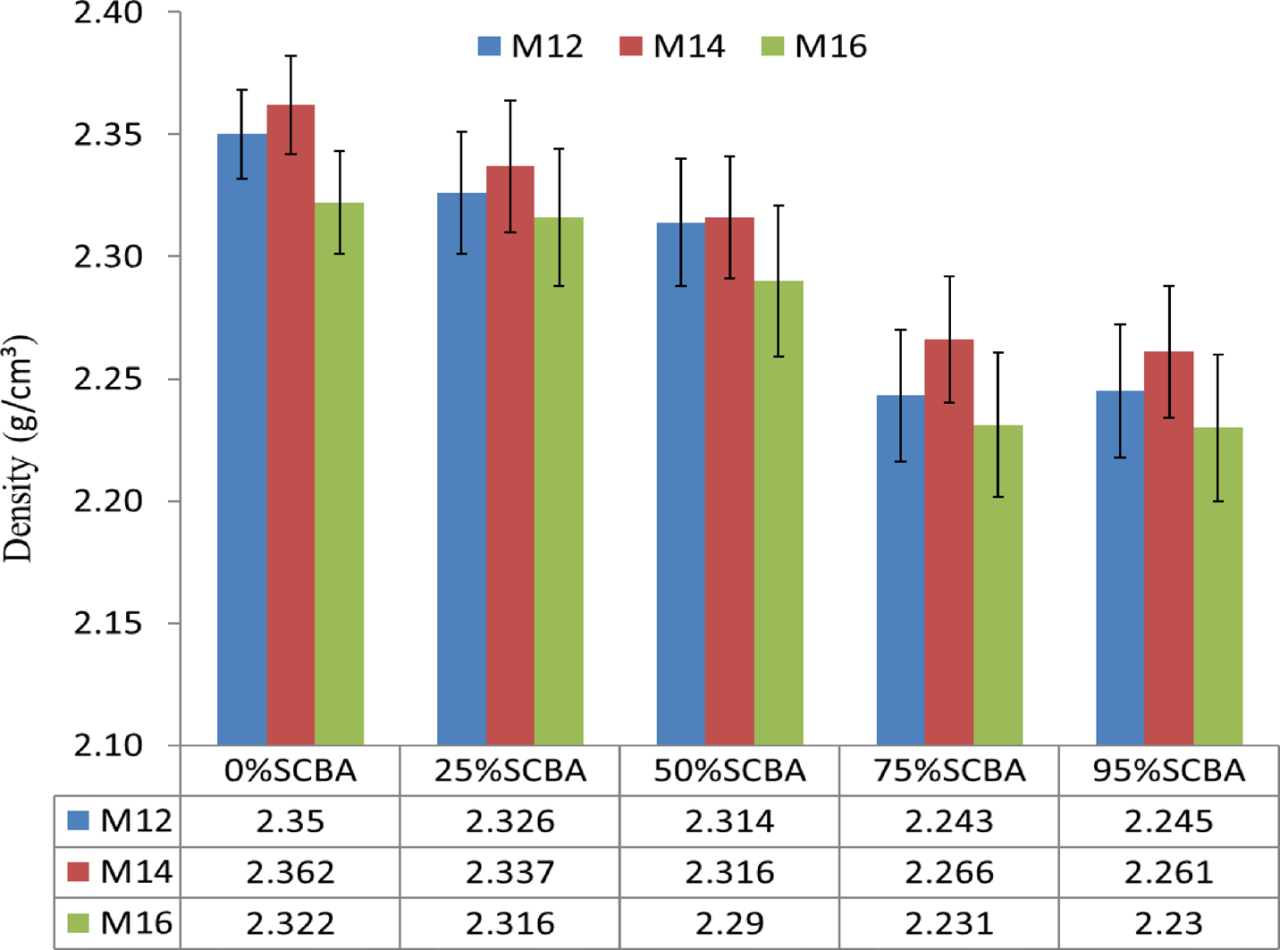
Density test results presented as mean values of three measurements (*n* = 3). Error bars represent standard deviation.

As presented in Fig. 22, the concrete density progressively decreased from a maximum of 2.362 g/cm³ (at 0% SCBA) to a minimum of 2.230 g/cm³ (at 95% SCBA) with increasing SCBA content. This reduction is primarily attributed to the porous nature and lower specific gravity of the SCBA particles, which increases internal micro-voids. Regarding the alkaline solution, the 14 M NaOH series showed the highest mean density across all replacement levels including the B25 mixture at 2.337 g/cm³ indicating an optimal alkalinity threshold for a more compact geopolymer matrix. Conversely, intermediate levels (25%–50%) showed a steady weight reduction, while the lowest density framework stabilized at the 75% and 95% replacement levels. The standard deviation of the triplicate density measurements ranged from 0.018 to 0.031 g/cm³. Two-way ANOVA showed significant effects of SCBA content (F(4,30) = 27.72, *p* < 0.001) and NaOH molarity (F(2,30) = 5.21, *p* = 0.011), whereas their interaction was not statistically significant (F(8,30) = 0.13, *p* = 0.997).

### Results of compressive strength test

**Fig. 23:**
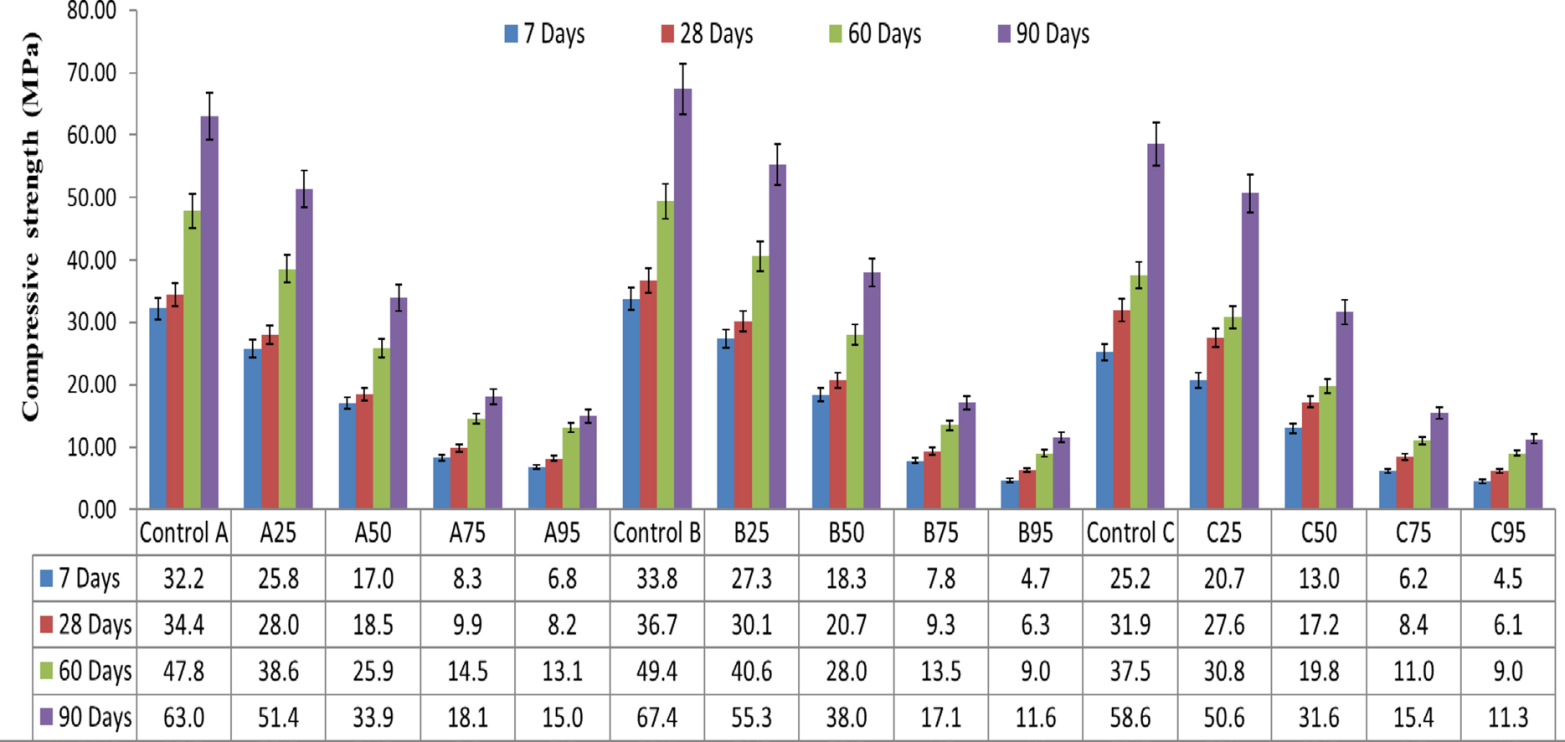
Mean compressive strength values obtained from three replicate specimens (*n* = 3). Error bars represent standard deviation.

As shown in Fig. 23, the compressive strength progressively decreased with increasing SCBA content across all curing ages, with values ranging from 4.5 to 67.4 MPa. This reduction is primarily attributed to the high SCBA content lowering the availability of reactive aluminosilicate phases, which yields a less homogeneous matrix with unreacted particles and internal voids. Similar structural trends regarding the impact of high SCBA replacement levels on matrix homogeneity were reported by V.N. Castaldelli^46^, albeit within a potassium-activated system. Conversely, the compressive strength continuously developed with curing age due to ongoing geopolymerization, showing a strength gain between 7 and 28 days ranging from 6.78% to 37.05%. Among the SCBA-containing mixtures, B25 exhibited the highest compressive strength, achieving a 28-day compressive strength of 30.13 MPa. This behavior stems from the favorable balance between fly ash and SCBA, which enhances the dissolution of aluminosilicate species and promotes a cohesive geopolymeric network.

Regarding the alkaline activator, increasing the NaOH molarity from 12 M to 14 M enhanced strength by facilitating precursor dissolution and binder formation. However, a further increase to 16 M lowered the strength, suggesting that excessive hydroxyl concentrations altered the reaction mechanism and hindered a uniformly bonded framework, consistent with the findings of Vo^16^. The standard deviation of the triplicate compressive strength measurements ranged from 0.30 to 4.05 MPa. Three-way ANOVA showed significant effects of SCBA content (F(4,120) = 2776.71, *p* < 0.001), NaOH molarity (F(2,120) = 117.63, *p* < 0.001), and curing age (F(3,120) = 1129.18, *p* < 0.001). All two-way interactions were significant (*p* < 0.001), whereas the three-way interaction was not statistically significant (F(24,120) = 0.90, *p* = 0.601).

### Results of indirect split tensile test

**Fig. 24:**
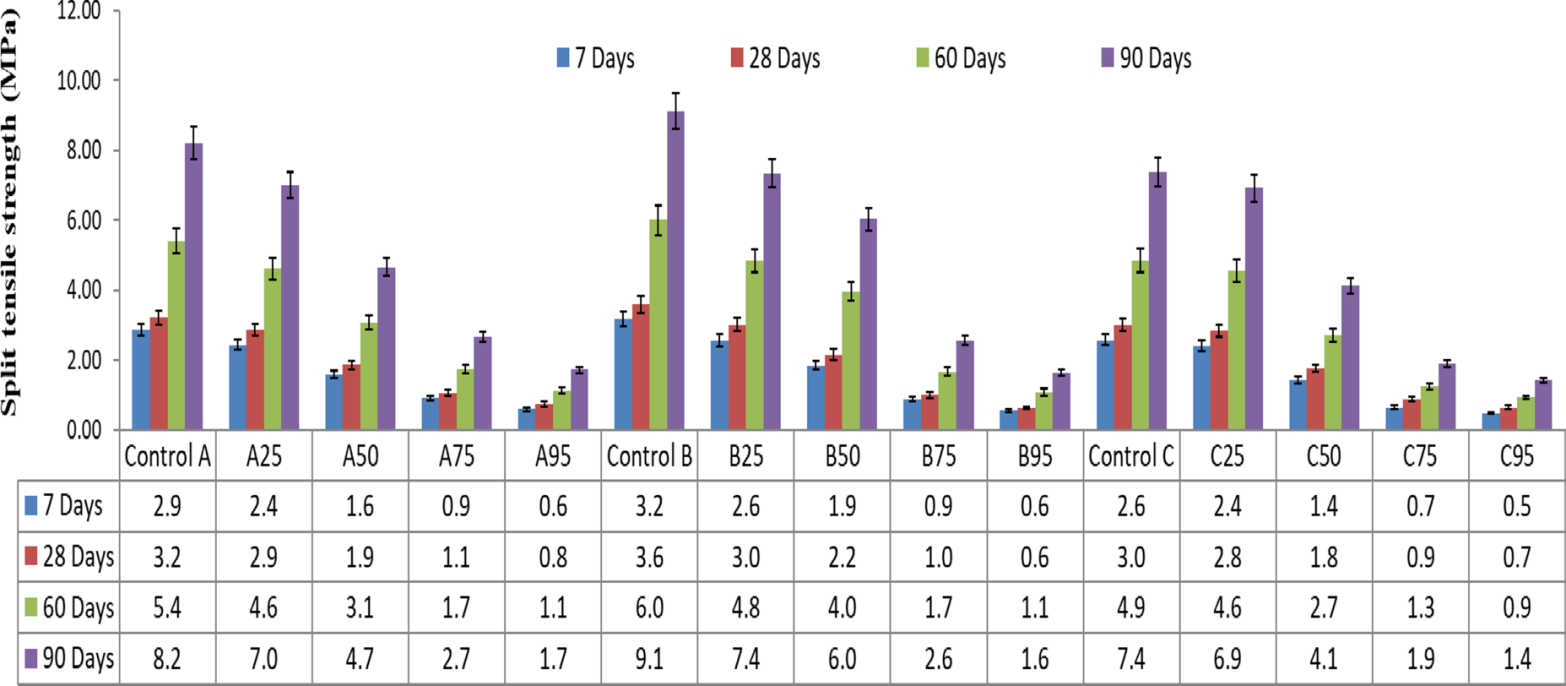
Mean splitting tensile strength values obtained from three replicate specimens (*n* = 3). Error bars represent standard deviation.

The splitting tensile strength results presented in Fig. 24 indicate a progressive reduction with increasing SCBA content across all curing ages, with values ranging from 0.488 to 9.12 MPa. Similar to the compressive strength trends, the incorporation of high SCBA contents adversely affected the matrix integrity, leading to a lower tensile capacity. Conversely, the tensile strength continuously developed with curing age, exhibiting strength gains between 12.19% and 34.30% from 7 to 28 days, which reflects the ongoing progression of the geopolymerization reactions. %. Among the SCBA-containing mixtures, B25 was confirmed the highest mixture, achieving a splitting tensile strength of 3.018 MPa at 28 days.

Regarding the alkaline activator effect, increasing the NaOH molarity enhanced the tensile performance up to 14 M, whereas a further increase to 16 M induced a strength reduction, a behavior that is highly consistent with the findings of Rihan^18^. The standard deviation of the triplicate measurements ranged from 0.030 to 0.506 MPa. Three-way ANOVA showed significant effects of SCBA content (F(4,120) = 2276.61, *p* < 0.001), NaOH molarity (F(2,120) = 95.96, *p* < 0.001), and curing age (F(3,120) = 1990.14, *p* < 0.001). All two-way interactions were statistically significant (*p* < 0.001), whereas the three-way interaction was not statistically significant (F(24,120) = 1.58, *p* = 0.058).

### Results of the Schmidt hammer test

The Schmidt hammer test was conducted at 28 days to evaluate the relationship between surface hardness and compressive strength of the investigated GPC mixtures. The average rebound numbers were measured, and the corresponding estimated compressive strengths were obtained using the manufacturer’s Schmidt hammer calibration curve. The calibration curve used in this study was the standard conversion chart supplied by the Schmidt hammer manufacturer for conventional concrete. It was used solely to obtain comparative strength estimates and was not developed specifically for geopolymer concrete. The estimated strengths together with the experimentally measured compressive strengths are presented in Table 9. The estimated compressive strengths obtained from the Schmidt hammer calibration (f_ch)_ generally followed the same trend as the experimentally measured compressive strengths (f_cu_), decreasing with increasing SCBA replacement level. The resulting empirical relationship is expressed by Eq. (8).

8 $$f_{{cu}} = {\text{ }}0.0096f_{{ch}}^{{\;2}} + {\text{ }}0.1039{\text{ }}f_{{ch}} + {\text{ }}5.8077$$

The regression yielded a coefficient of determination of R² = 0.997 with a statistically significant p-value (*p* < 0.001). However, this empirical relationship was developed using the same experimental dataset and was not independently validated. Therefore, it should be interpreted only as a dataset-specific correlation and not as a universally applicable predictive calibration model for geopolymer concrete.

**Fig. 25:**
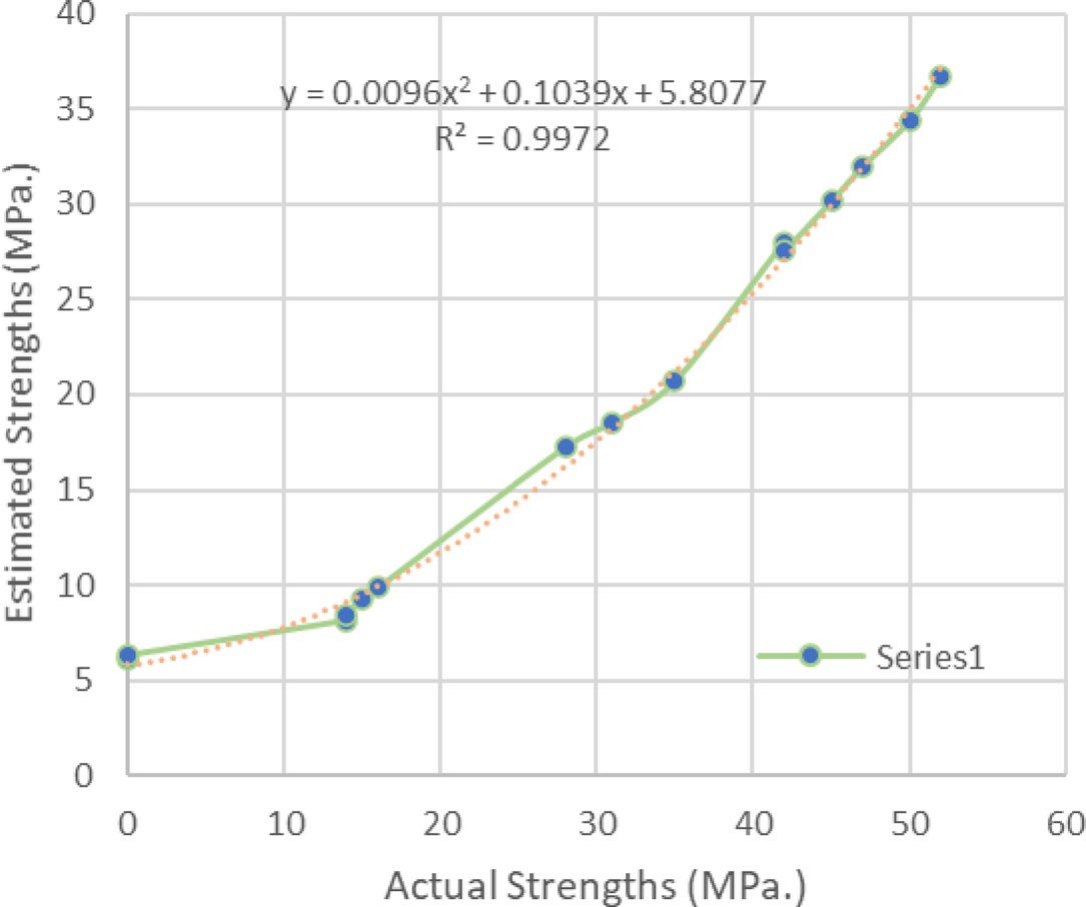
Relationship between the estimated compressive strengths obtained from the Schmidt hammer calibration and the experimentally measured 28-day compressive strengths of the investigated geopolymer concrete mixtures.

The results indicate a strong association between the Schmidt hammer estimates and the experimentally measured compressive strengths within the investigated dataset; however, this association does not imply numerical agreement or independent predictive validity. Differences between the estimated and measured strengths may arise because the Schmidt hammer primarily evaluates surface hardness and its standard conversion chart was developed for conventional concrete rather than geopolymer systems. Accordingly, the Schmidt hammer should be regarded as a complementary non-destructive method for comparative assessment and preliminary strength estimation, while direct compression testing remains the reference method for determining compressive strength. Figure 25 illustrates the relationship between the Schmidt hammer-estimated strengths and the experimentally measured compressive strengths of the investigated geopolymer concrete mixtures.

**Table 9:** Average rebound numbers and corresponding 28-day compressive strengths of the investigated geopolymer concrete mixtures.

Symbol	Average rebound number	Estimated compressive strength from Schmidt hammer calibration (MPa)	Actual compressive strength (MPa)
Control A	44.12	50	34.35
A25	38.48	42	28
A50	32.15	31	18.48
A75	21.46	16	9.86
A95	20.02	14	8.15
Control B	48.86	52	36.7
B25	39.92	45	30.13
B50	34.61	35	20.69
B75	20.52	15	9.3
B95	15.37	-	6.3
Control C	41.19	47	31.94
C25	38.62	42	27.56
C50	29.79	28	17.22
C75	19.93	14	8.41
C95	11.37	-	6.14

The rebound numbers recorded for mixtures B95 and C95 were below the valid calibration range of the Schmidt hammer conversion chart. Therefore, no corresponding estimated compressive strengths could be obtained.

### Results of sorptivity test

**Fig. 26:**
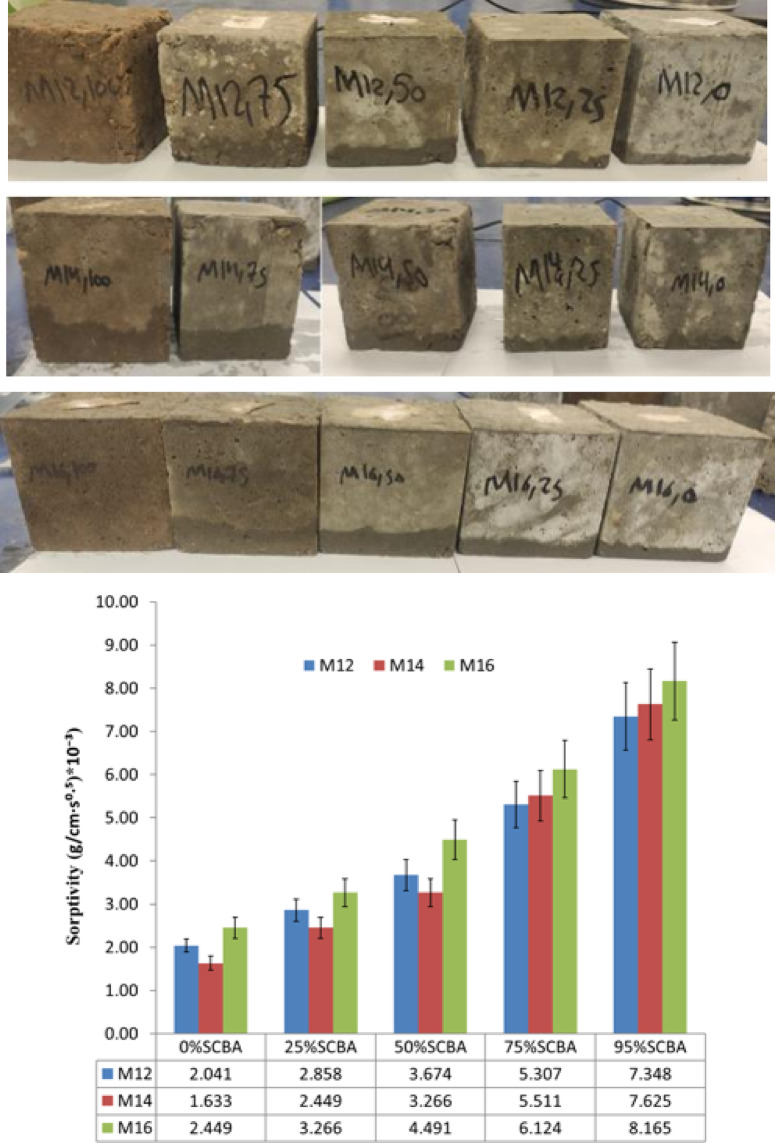
The sorptivity test results.

At 28 days, the sorptivity values ranged from 1.633 × 10⁻³ to 8.165 × 10⁻³ g/cm·s⁰·⁵, as illustrated in Fig. 26, with standard deviation values ranging from 0.15 × 10⁻³ to 0.90 × 10⁻³ g/cm·s⁰·⁵. Sorptivity increased with increasing SCBA content and generally decreased with increasing density and compressive strength, indicating the formation of a denser and less permeable geopolymer matrix. The lower sorptivity exhibited by the 14 M mixtures can be attributed to enhanced aluminosilicate dissolution and more effective geopolymerization, resulting in greater matrix densification, reduced pore connectivity, and fewer capillary voids. In contrast, high SCBA replacement levels increased porosity and interconnected voids due to the porous nature and lower reactivity of SCBA, leading to higher water absorption rates. The standard deviation of the triplicate measurements ranged from 0.15 × 10⁻³ to 0.90 × 10⁻³ g/cm·s⁰·⁵. Two-way ANOVA showed significant effects of SCBA content (F(4,30) = 178.74, *p* < 0.001) and NaOH molarity (F(2,30) = 10.42, *p* < 0.001), whereas their interaction was not statistically significant (F(8,30) = 0.47, *p* = 0.865).

### Results of elevated temperature tests

#### Visual changes

**Fig. 27:**
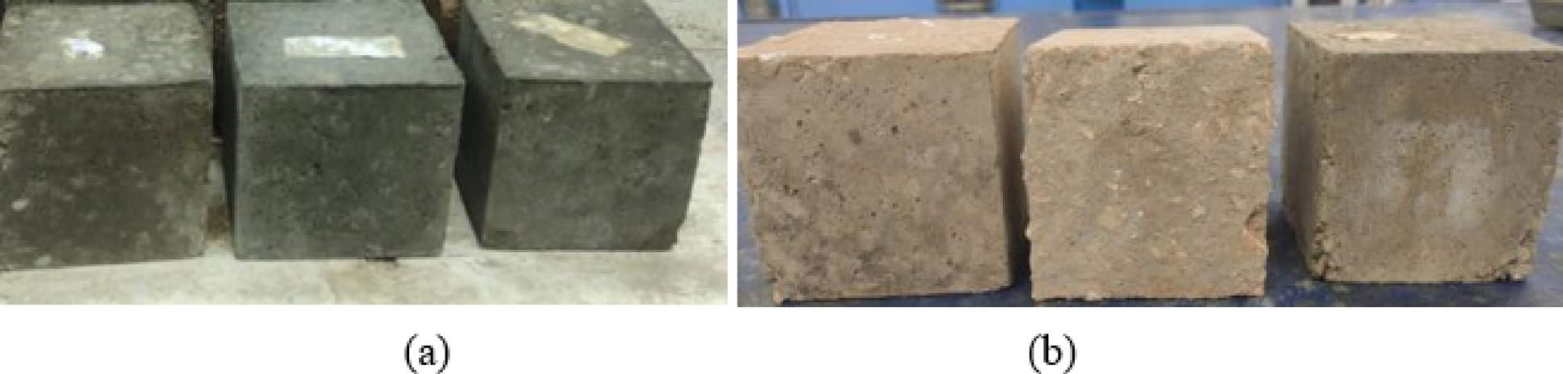
Visual changes for specimens; (**a**) before and (**b**) after heat exposure.

As shown in Fig. 27, no noticeable visual changes were observed at 100 °C, which can be attributed to the evaporation of free water from the geopolymer matrix. At 200 °C, slight color changes appeared as a result of dehydration processes. Exposure to 300 °C led to the initiation of visible cracks due to thermal stresses and internal vapor pressure. At higher temperatures (400–500 °C), extensive cracking and surface deterioration were observed, indicating progressive degradation of the geopolymeric gel structure and consequent loss of mechanical integrity.

#### Mass loss

**Fig. 28:**
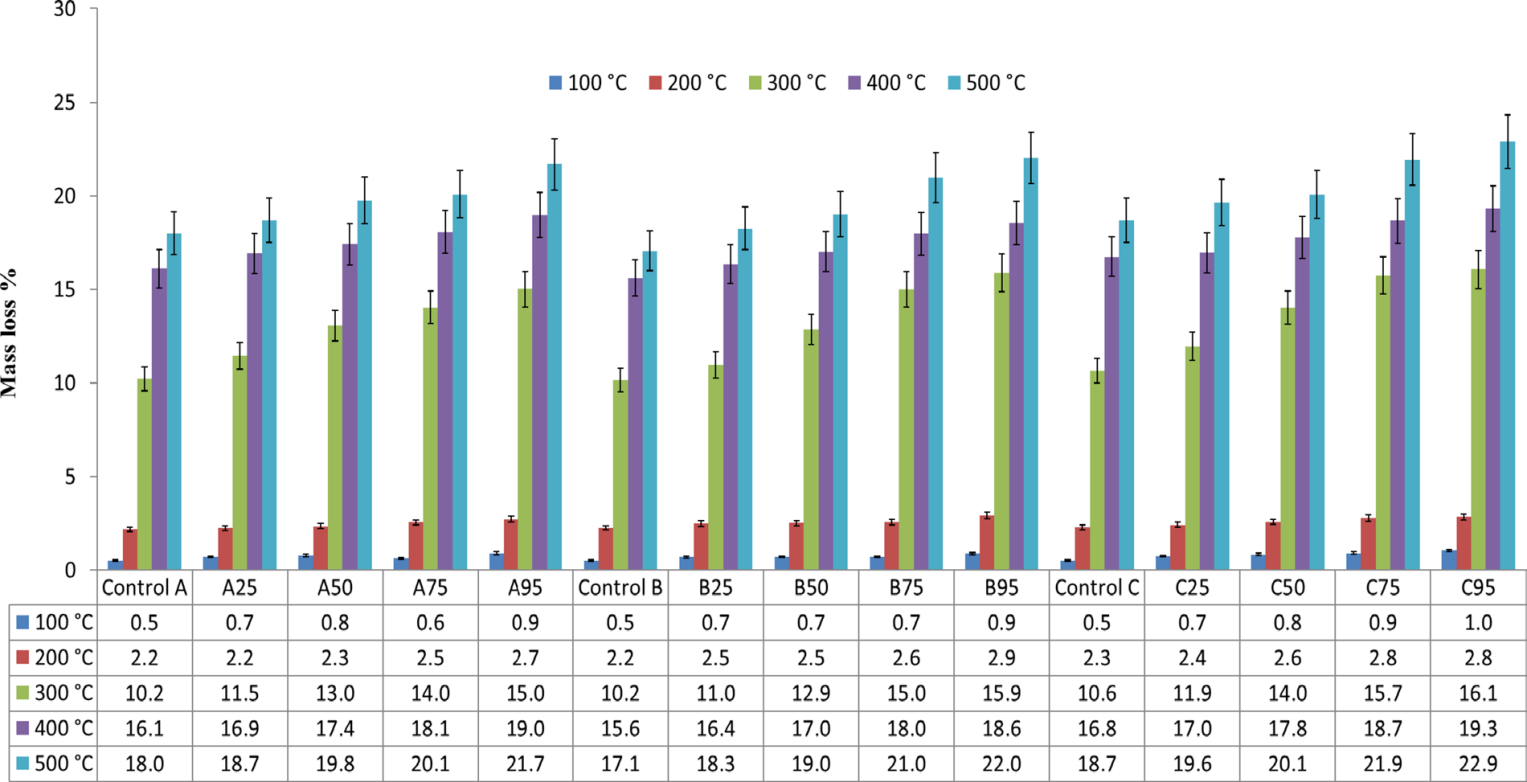
Mean mass loss values after exposure to elevated temperatures (*n* = 3). Error bars represent standard deviation.

Mass loss increased progressively with increasing temperature, ranging from 0.508 to 1.031% at 100 °C and 2.17–2.83% at 200 °C, before rising substantially to 10.15–17.52%, 15.61–19.31%, and 17.99–22.90% at 300, 400, and 500 °C, respectively as indicated Fig. 28. The standard deviation values ranged between 0.045 and 1.44%, indicating acceptable repeatability of the measurements. The initial mass loss was primarily associated with the evaporation of free and physically bound water, whereas the higher losses at elevated temperatures resulted from geopolymer gel dehydration, microcrack development, and progressive matrix deterioration. Mixtures containing higher SCBA contents exhibited greater mass loss due to their more porous structure and higher moisture retention capacity. The standard deviation of the triplicate measurements ranged from 0.045 to 1.440%. Three-way ANOVA showed significant effects of temperature (F(4,150) = 4839.10, *p* < 0.001), SCBA content (F(4,150) = 74.72, *p* < 0.001), and NaOH molarity (F(2,150) = 10.48, *p* < 0.001). The interaction between SCBA content and temperature was also significant (F(16,150) = 10.92, *p* < 0.001), whereas the remaining interactions were not statistically significant (*p* > 0.05).

#### Compressive strength retention

**Fig. 29:**
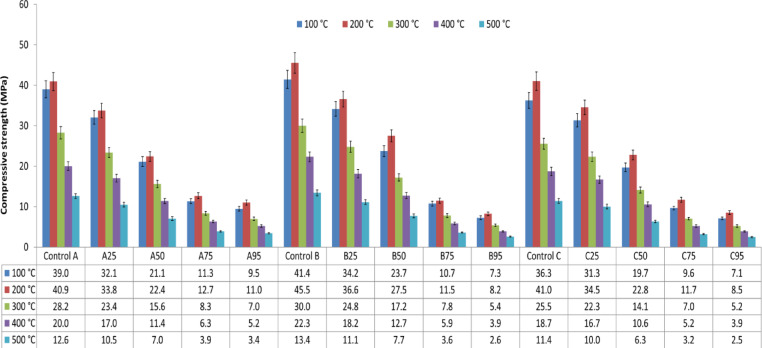
Compressive strength of geopolymer concrete mixtures after exposure to elevated temperatures.

Figure 29 illustrates the influence of elevated temperature exposure on the compressive strength of the investigated geopolymer concrete mixtures. Characteristically, initial thermal exposure between 100 °C and 200 °C induced noticeable strength gains ranging from 12.92% to 39.30%, a phenomenon that was particularly pronounced in mixtures incorporating higher SCBA contents. This behavior is attributed to the evaporation of physically bound water and the continued geopolymerization of partially reacted aluminosilicate species, leading to the subsequent formation of binding gels and a denser microstructure.

However, beyond 300 °C, the compressive strength exhibited a distinct decline of 14.53%–20.05%, signifying the initiation of thermal microcracking and localized matrix deterioration. Further temperature increases to 400 °C and 500 °C caused substantial strength losses of 35.90%–41.74% and 58.28%–64.25%, respectively. This severe mitigation stems from the structural dehydration of the geopolymeric gels, the structural breakdown of the aluminosilicate network, increased pore connectivity, and extensive crack propagation throughout the matrix.

From a material composition perspective, Mixtures containing higher SCBA contents generally exhibited higher strength retention after exposure to elevated temperatures. However, owing to their lower initial compressive strengths, their absolute residual strengths remained below those of mixtures with lower SCBA replacement levels. Therefore, the thermal performance should be evaluated using both the residual compressive strength and the percentage strength retention. When comparing the specific mixture groups, the 14 M NaOH series (Group B) systematically maintained higher residual compressive strength values across all thermal stages compared to the 12 M (Group A) and 16 M (Group C) counterparts. Notably, the B25 mixture demonstrated an exceptional capacity to withstand thermal degradation, preserving a substantial portion of its structural integrity up to 300 °C before experiencing accelerated loss at higher thresholds. Although the B75 and C95 mixtures retained a higher percentage of their original compressive strength, the Control and B25 mixtures maintained higher absolute residual compressive strengths after thermal exposure, indicating superior residual load-carrying capacity.

In contrast, increasing the NaOH molarity beyond 14 M (as observed in Group C) resulted in greater strength deterioration after heating. This trend is likely driven by the highly dense yet brittle nature of the high-molarity matrix, rendering it more susceptible to thermally induced structural stresses and internal vapor pressure accumulation. Among all investigated designs incorporating SCBA, the B25 mixture was identified as the optimum design because a 25% SCBA replacement level provides the ideal reactive SiO_2_/Al_2_O_3_ ratio, which maximizes binder gel formation without undermining matrix uniformity. To visually verify this behavior at the microstructural level, Scanning Electron Microscopy (SEM) was performed. The standard deviation of the triplicate residual compressive strength measurements ranged from 0.15 to 2.55 MPa. Three-way ANOVA showed significant effects of SCBA content (F(4,150) = 3645.75, *p* < 0.001), NaOH molarity (F(2,150) = 59.56, *p* < 0.001), and exposure temperature (F(4,150) = 1976.54, *p* < 0.001), whereas the three-way interaction was not statistically significant (F(32,150) = 0.87, *p* = 0.673).

#### Tensile strength retention

**Fig. 30:**
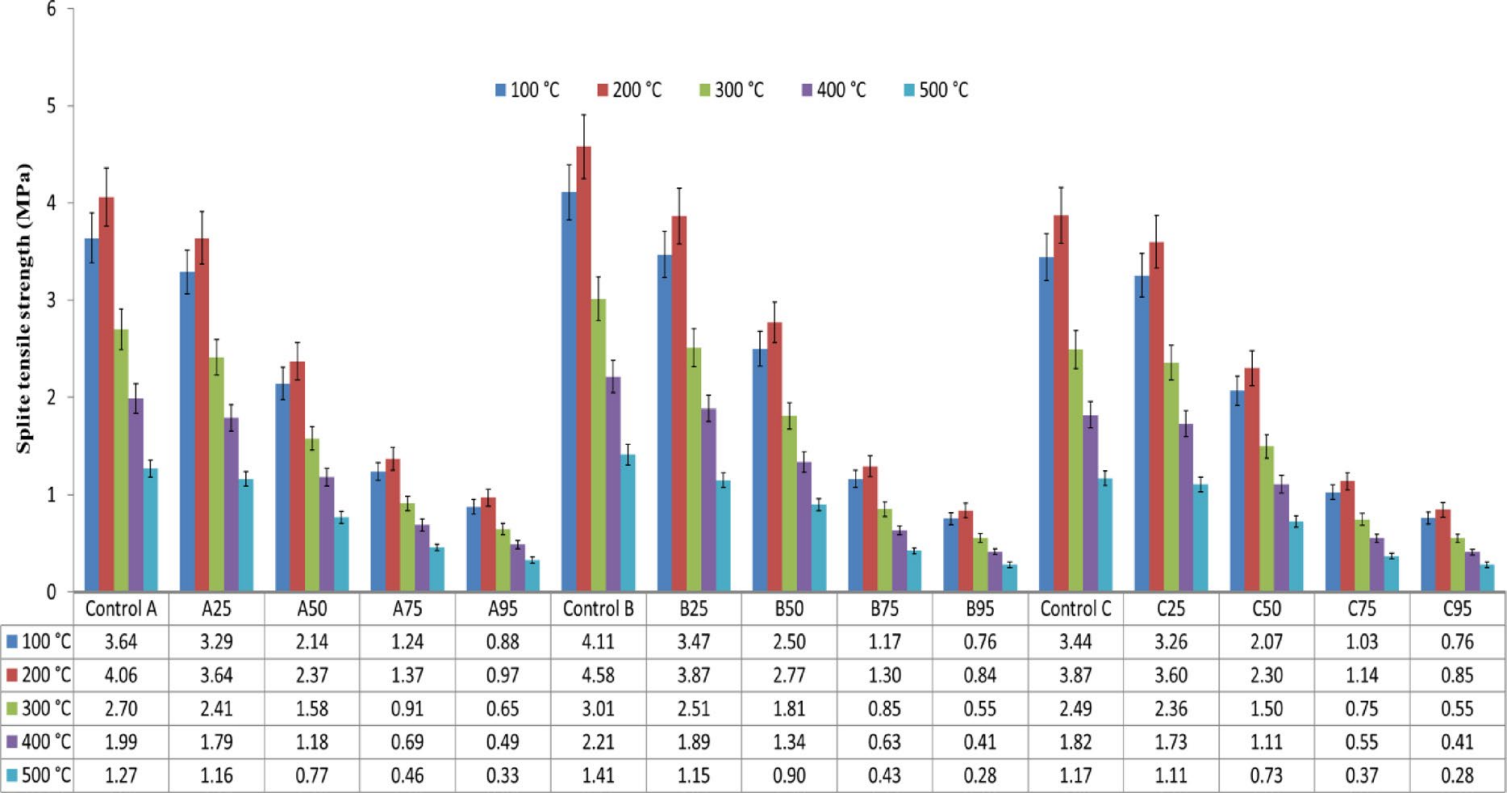
Mean residual splitting tensile strength after exposure to elevated temperatures (*n* = 3). Error bars represent standard deviation.

Figure 30 presents the variation in the splitting tensile strength of the geopolymer concrete mixtures after exposure to elevated temperatures. Characteristically, exposure to thermal loads between 100 °C and 200 °C induced a noticeable strength enhancement across all investigated mixtures, with the peak tensile capacity recorded for the Control B mixture reaching 4.58 MPa at 200 °C. Beyond 300 °C, however, a severe and progressive deterioration in tensile performance was observed, with the lowest residual strengths systematically recorded at 500 °C (ranging from 0.28 to 1.41 MPa).

The underlying physical and chemical mechanisms governing this temperature-dependent behavior closely mirror those identified during the compressive strength testing. The initial strength enhancement at lower thermal stages (100–200 °C) is attributed to accelerated geopolymerization driven by the internal autoclaving effect and the further dissolution of unreacted binder phases. Conversely, the profound strength mitigation recorded at higher thermal thresholds (300–500 °C) stems directly from structural geopolymer gel dehydration, thermal microcrack propagation, and differential thermal expansion within the matrix. Notably, the 14 M NaOH series (Group B) consistently maintained superior residual tensile strength at elevated temperatures compared to the 12 M (Group A) and 16 M (Group C) series, with the B25 mixture exhibiting a robust performance of 3.87 MPa at 200 °C before shifting into a controlled degradation. Although mixtures with higher SCBA contents sometimes retained a larger percentage of their initial tensile strength, their absolute residual tensile strengths remained lower than those of the control and lower-SCBA mixtures. Therefore, both percentage retention and absolute residual tensile strength should be considered when evaluating thermal performance. The standard deviation of the triplicate residual splitting tensile strength measurements ranged from 0.03 to 0.33 MPa. Three-way ANOVA showed significant effects of SCBA content (F(4,150) = 1862.65, *p* < 0.001), NaOH molarity (F(2,150) = 40.67, *p* < 0.001), and exposure temperature (F(4,150) = 1010.99, *p* < 0.001). The interactions between SCBA content and NaOH molarity and between SCBA content and temperature were also significant (*p* < 0.001), whereas the remaining interactions were not statistically significant (*p* > 0.05).

### Results of microstructure tests

#### SEM analysis of the B25 mixture at normal temperature

**Fig. 31:**
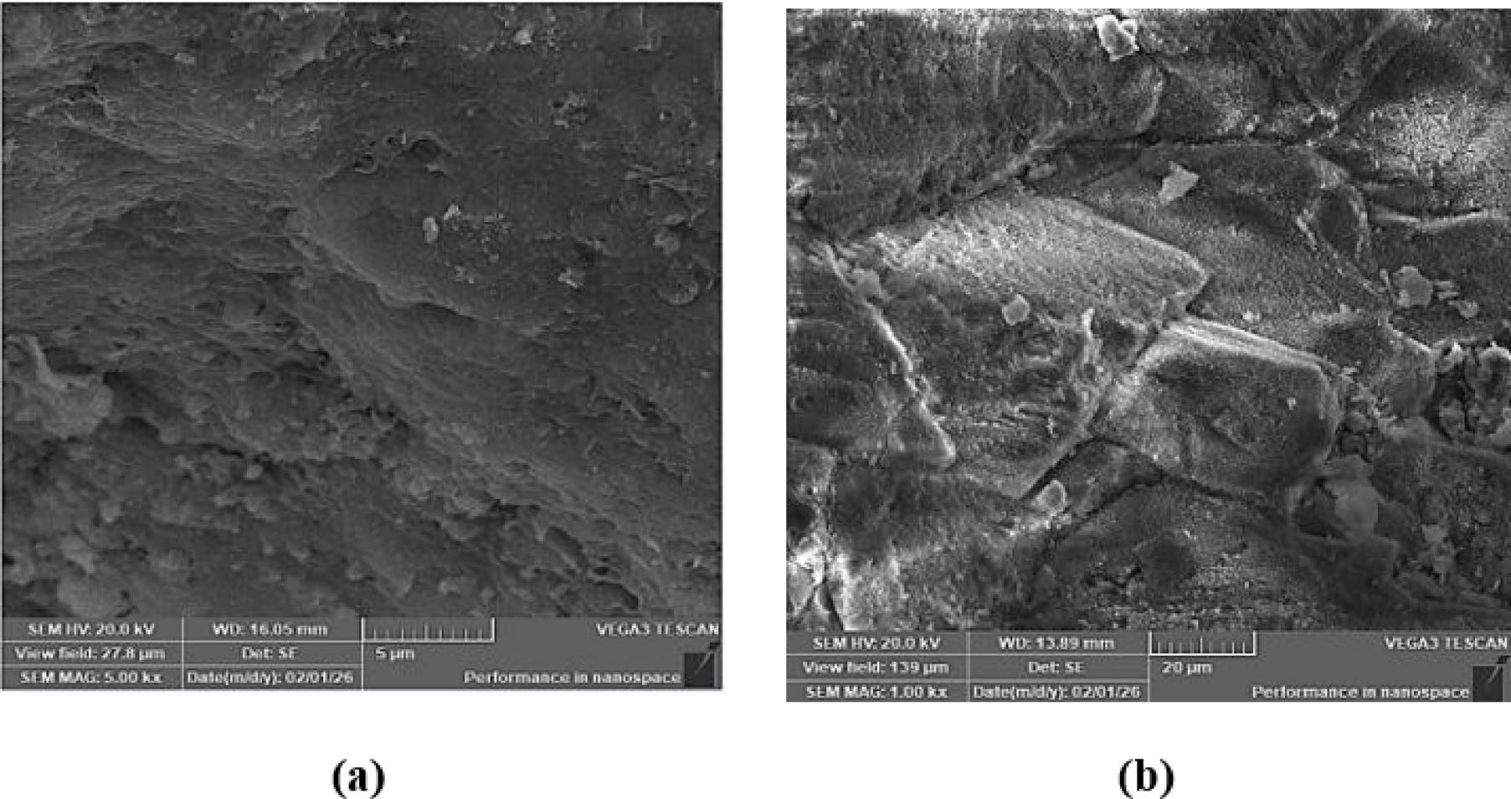
SEM micrographs of the B25 mixture at different magnifications.

Figure 31 presents the SEM micrographs of the B25 mixture 28 days of ambient curing at two different magnification levels (1.00 kx and 5.00 kx). At the higher magnification of 5.00 kx (Fig. 31a), the microstructure exhibits a highly dense, compacted, and well-developed geopolymeric gel framework characterized by a distinct layered or continuous flaky morphology. This dense matrix architecture reveals an exceptional reduction in capillary porosity, directly explaining the comparatively high compressive strength and low sorptivity among the SCBA-containing mixtures.

Simultaneously, the wider field of view captured at 1.00 kx magnification (Fig. 31b) demonstrates outstanding structural homogeneity throughout the binder matrix. The micrograph reveals a well-interconnected network featuring only a minor presence of partially reacted or fully embedded SCBA particles, indicating that the 14 M NaOH activator effectively facilitated a high degree of aluminosilicate dissolution. Furthermore, the absence of extensive microcracking and interconnected voids underlines the stable volumetric nature of the matrix during ambient curing. These observations solidly support the conclusion that a 25% SCBA replacement level combined with 14 M NaOH provides a favorable balance between precursor reactivity and geopolymeric gel development, resulting in a compact, homogeneous, and mechanically efficient microstructure.

#### SEM analysis for different mixtures after exposure to elevated temperatures

To thoroughly understand the coupled effects of thermal exposure and precursor composition on the macroscopic mechanical degradation, an advanced microstructural and chemical investigation via SEM-EDX was executed. This phase of analysis focuses on tracking the evolution of binder morphology, microcrack kinetics, and elemental distribution profiles within two contrasting systems: the baseline Control A mixture exposed to a moderate thermal load (300 °C), and the high-ash B75 geopolymeric matrix subjected to severe thermal stress (500 °C). By evaluating these specific configurations, the direct correlation between chemical shifting (such as the Si/Al ratio) and physical fabric deterioration can be precisely mapped.

**Fig. 32:**
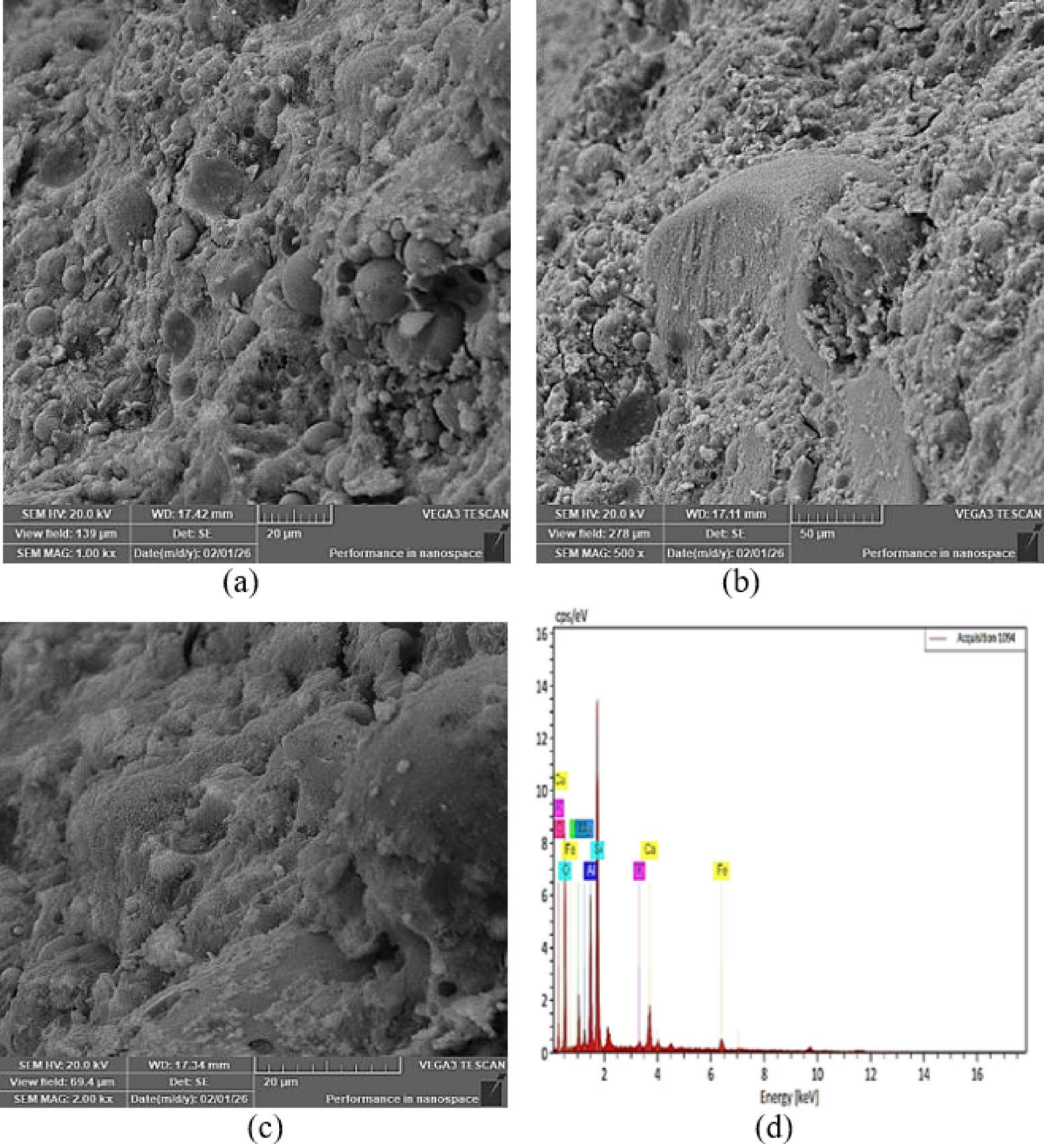

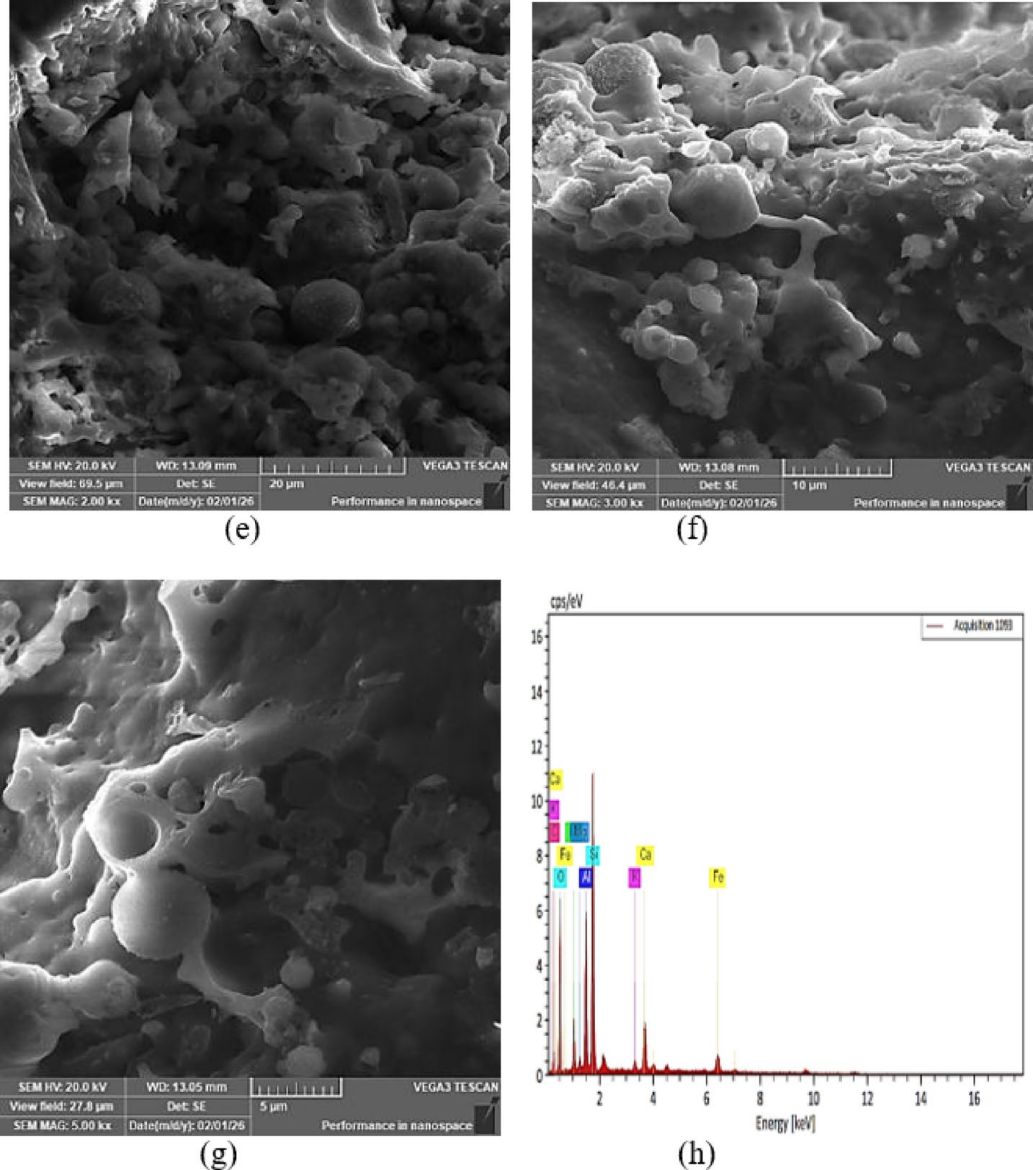
SEM-EDX of mix Control A, (**a**–**d**) at ambient temperature and (**e**–**h**) after heating at 300 °C.

SEM-EDX analysis was performed to investigate the microstructural evolution of the Control A matrix following thermal exposure. Under ambient conditions, the SEM micrographs (Fig. 32a and b) revealed a compact matrix with well-integrated reaction products and limited visible voids, reflecting effective precursor dissolution and reaction progress. The corresponding EDX results (Fig. 32c and d) indicated an initial Si/Al ratio of approximately 2.21, which coincided with the highest baseline compressive strength of 34.35 MPa.

After exposure to 300 °C (Fig. 32e and h), the microstructure became less continuous, marked by the appearance of fine thermal cracks and increased separation between the reaction products. These morphological changes were accompanied by a reduction in the Si/Al ratio to approximately 1.85, suggesting alterations in the chemical balance of the binding phases due to partial desiccation. The combined effects of moisture release, partial breakdown of reaction products, and the development of internal defects weakened the matrix continuity and reduced its capacity to resist applied loads, resulting in a decrease in compressive strength to 28.25 MPa. The observed microstructural deterioration demonstrates the strong relationship between thermal exposure, changes in binder chemistry, and the resulting loss of mechanical performance^13,14,16–26,46,47^.

**Fig. 33:**
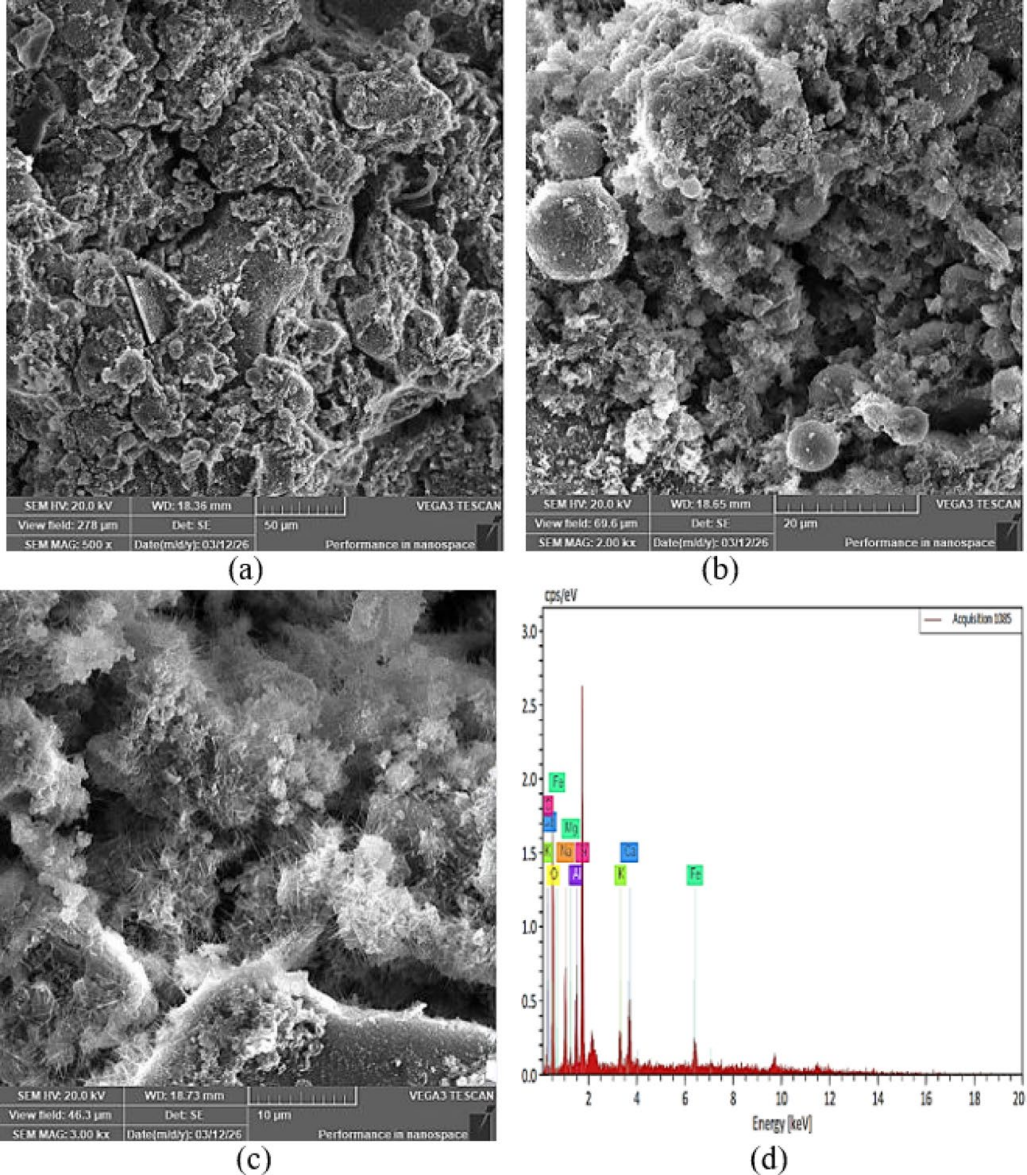

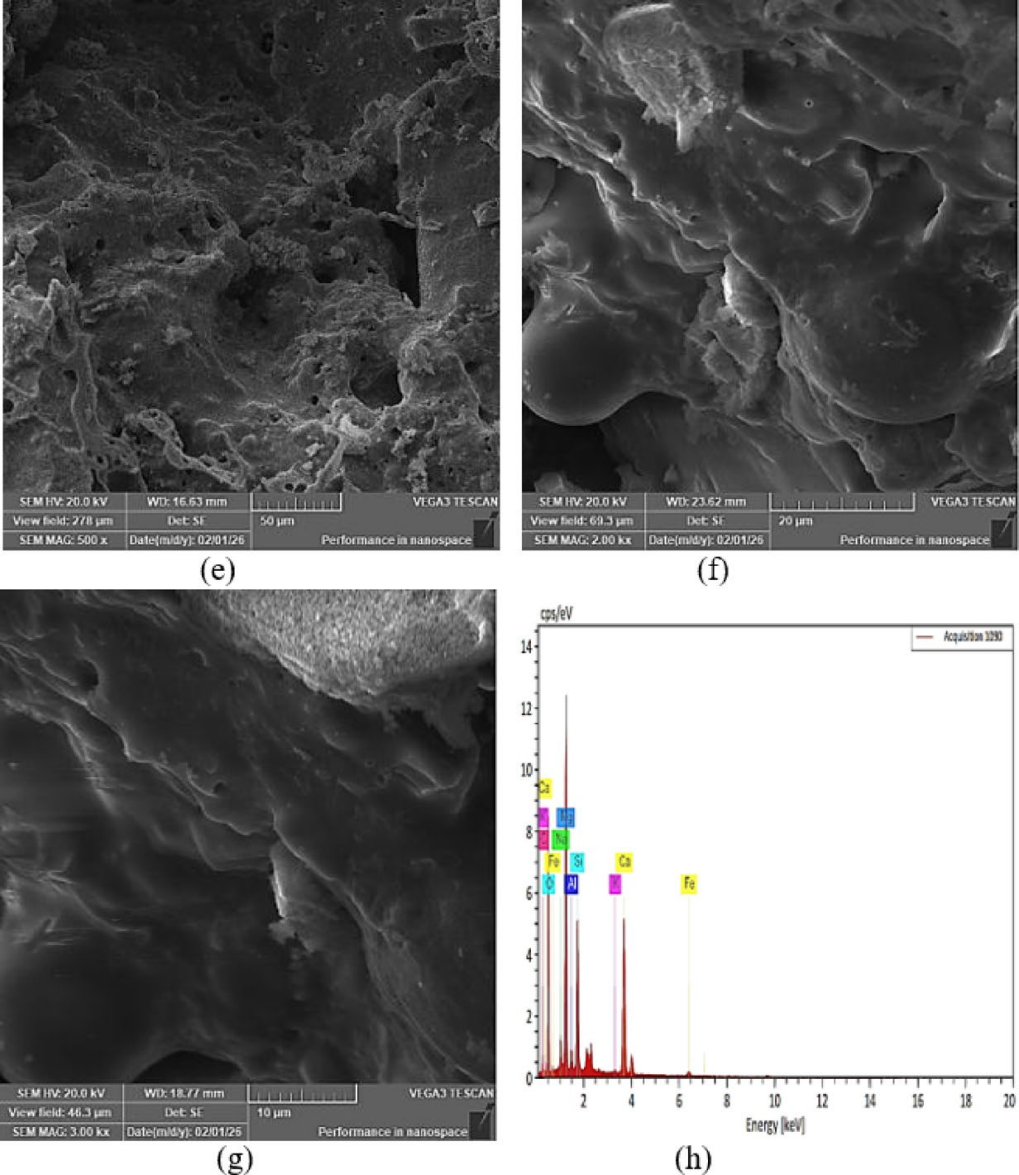
SEM-EDX of B75, (**a**–**d**) at ambient temperature and (**e**–**h**) after heating at 500 °C.

Figure 33 presents the SEM-EDX results of the high-ash B75 mixture under ambient conditions and after exposure to a severe thermal threshold of 500 °C. Before thermal exposure (Fig. 33a and d), the microstructure consisted of a relatively coherent matrix containing geopolymeric reaction products together with a limited amount of unreacted precursor particles. The corresponding EDX analysis yielded a Si/Al ratio of 3.59, which was associated with an ambient compressive strength of 9.30 MPa.

After exposure to 500 °C (Fig. 33e and h), the micrographs revealed pronounced cracking, larger coalesced voids, and extensive fragmentation of the matrix, indicating substantial structural damage caused by the high thermal loading. Interestingly, EDX analysis showed an increase in the Si/Al ratio to approximately 6.35, accompanied by a noticeable reduction in the aluminum content and the presence of secondary Mg- and Ca-bearing phases. This chemical shift suggests that high-temperature exposure altered the original composition of the binding phases and modified their distribution within the matrix. As a result, the continuity of the hardened structure was disrupted, leading to a marked reduction in compressive strength to 3.61 MPa. These observations critically demonstrate that the physical microstructural condition of the matrix, rather than the Si/Al ratio alone, governs the residual mechanical performance after severe thermal exposure^25^.

In summary, the comparative SEM–EDX evidence reveals a transition from localized physical microcracking in the unreplaced matrix at 300 °C to severe chemical decomposition and structural disintegration in the high-ash framework at 500 °C, establishing that both thermal severity and ash dosage critically dictate the residual performance threshold of geopolymer composites.

#### Effect of alkaline solution molarity on microstructural integrity

To further elucidate the chemical and physical boundaries of activator concentrations, a comparative microstructural assessment was conducted to evaluate the influence of NaOH molarity. While the concentration of the alkaline activator is crucial for initiating the dissolution of aluminosilicate precursors, excessive or insufficient alkalinity can fundamentally alter the reaction kinetics, gel precipitation, and phase uniformity. This section contrasts the microstructural behavior of mixtures activated with 12 M (Group A) and 16 M (Group C) NaOH against the optimized 14 M (Group B) system to highlight the mechanisms governing matrix compactification and elemental shifting.

#### Comparative analysis of 12 M vs. 16 M NaOH activation (A25 vs. C25)

**Fig. 34:**
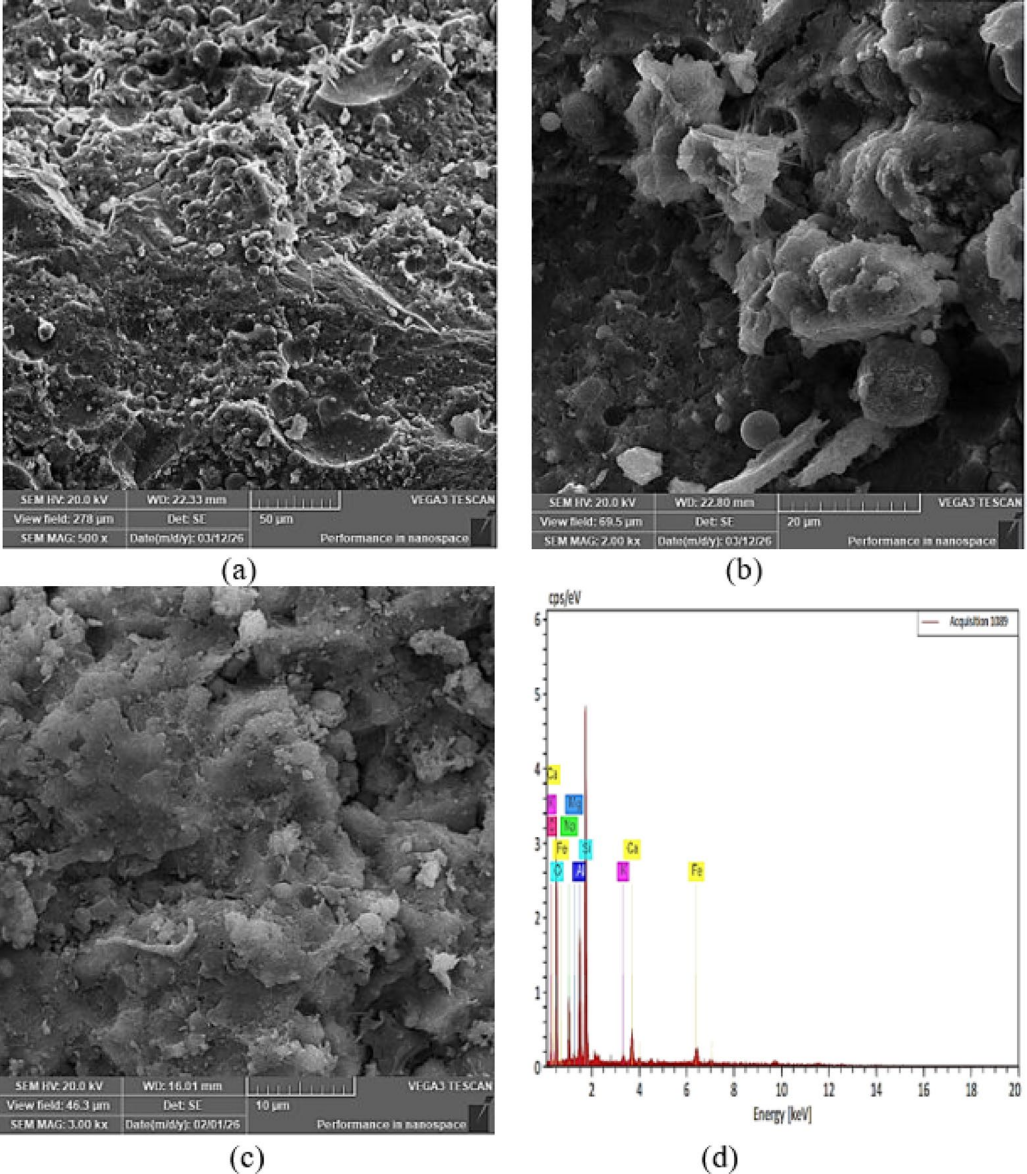

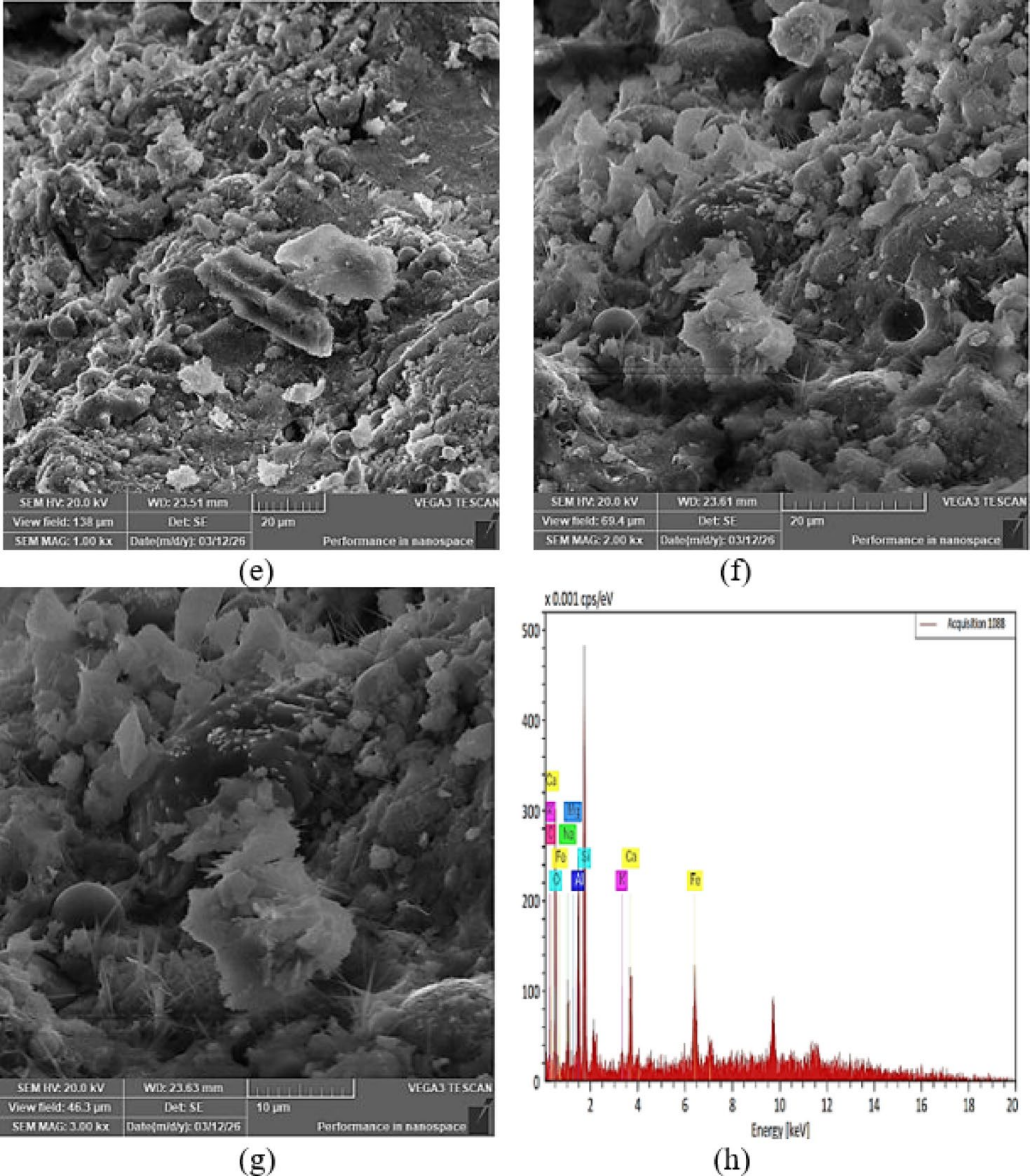
SEM-EDX analysis; (**a**–**d**) for A25 and **e**–**h**) for C25.

Figure 34 compares the SEM–EDX results of the A25 and C25 mixtures to systematically assess the influence of NaOH molarity. At a lower molarity of 12 M (Fig. 34a and d), the microstructure appeared relatively heterogeneous and porous, showing loosely packed SCBA particles. The corresponding EDX analysis indicated a high Si/Al ratio of approximately 2.55, which was associated with a compressive strength of 28.0 MPa due to incomplete precursor activation.

Conversely, increasing the activator molarity to 16 M (Fig. 34e and h) resulted in a visually more compact matrix; however, localized discontinuities, microstructural irregularities, and micro-cracks remained evident. Under this highly alkaline environment, the Si/Al ratio decreased to approximately 1.94, while the compressive strength slightly declined to 27.6 MPa. These findings indicate that increasing the activator concentration beyond the optimum threshold does not necessarily improve the matrix quality. Excessive alkalinity accelerates the dissolution of aluminosilicate species prematurely and alters the gel nucleation rate, leading to a less uniform reaction structure.

Therefore, the superior performance of the B25 mixture confirms that 14 M NaOH provided the most optimal balance between precursor dissolution and geopolymeric gel development, yielding a denser, more cohesive binding matrix. Similar behavior was reported by França et al.^48^, who demonstrated that the geopolymerization of SCBA-based binders is highly dependent on alkali concentration, where the reaction kinetics and diffusion-controlled formation of aluminosilicate gels significantly dictate the resulting mechanical efficiency.

#### High-ash, high-alkalinity matrix (C75)

**Fig. 35:**
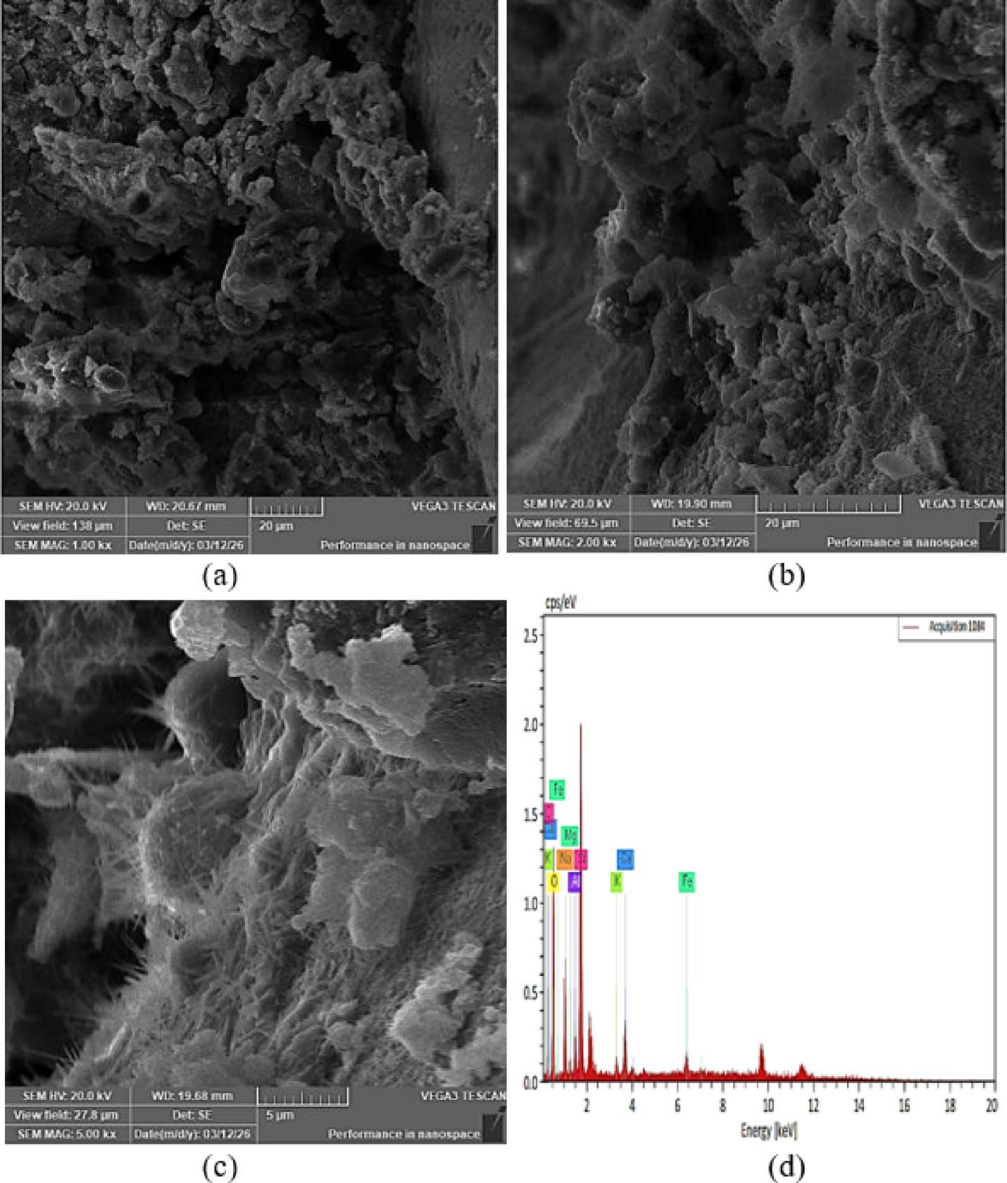
The SEM and EDX for C75 at ambient temperature.

Figure 35 illustrates the SEM–EDX results of the C75 mixture, representing the combined effects of high ash content and maximum alkalinity under ambient conditions. The micrographs and elemental spectra (Fig. 35a and d) reveal a highly porous, detached, and fragmented microstructure. This configuration yielded an excessively high Si/Al ratio of approximately 7.61 and a drastically low compressive strength of 8.41 MPa.

Compared with the optimized B25 mixture, the simultaneous introduction of higher SCBA replacement levels and excessive alkalinity (16 M) significantly undermined matrix integrity. This hostile chemical environment limited the formation of a well-connected, continuous geopolymeric network, leading to severe macro-pores and unreacted ash encapsulation that accumulated into a compromised mechanical performance.

In summary, the microstructural evidence gathered across different molarities establishes that while 12 M NaOH is insufficient to fully dissolve the source materials, a 16 M concentration induces a rapid, non-uniform precipitation that disrupts gel continuity. Consequently, a critical threshold of 14 M NaOH combined with 25% SCBA is microstructurally verified as the definitive optimum, successfully harmonizing chemical dissolution kinetics with dense physical matrix development.

#### Effect of SCBA replacement ratio

To establish a comprehensive baseline for the macro-mechanical fluctuations, it is crucial to analyze the progressive structural transitions induced by varying the Sugarcane Bagasse Ash (SCBA) replacement levels. Incorporating SCBA alters the reactive silica-to-alumina balance within the system, shifting the geopolymerization kinetics from a highly uniform matrix to a diffusion-controlled, multi-phase framework. This section systematically evaluates how increasing the ash dosage triggers physical network transitions, elemental redistribution, and porosity development.

Increasing the SCBA replacement level markedly affected both the microstructure and mechanical performance of the geopolymer mixtures. Initially, replacing fly ash with 25% SCBA reduced the compressive strength from 34.35 MPa for Control A to 28.0 MPa for A25, a trend primarily driven by a slightly less compact matrix and the presence of isolated, partially reacted particles.

**Fig. 36:**
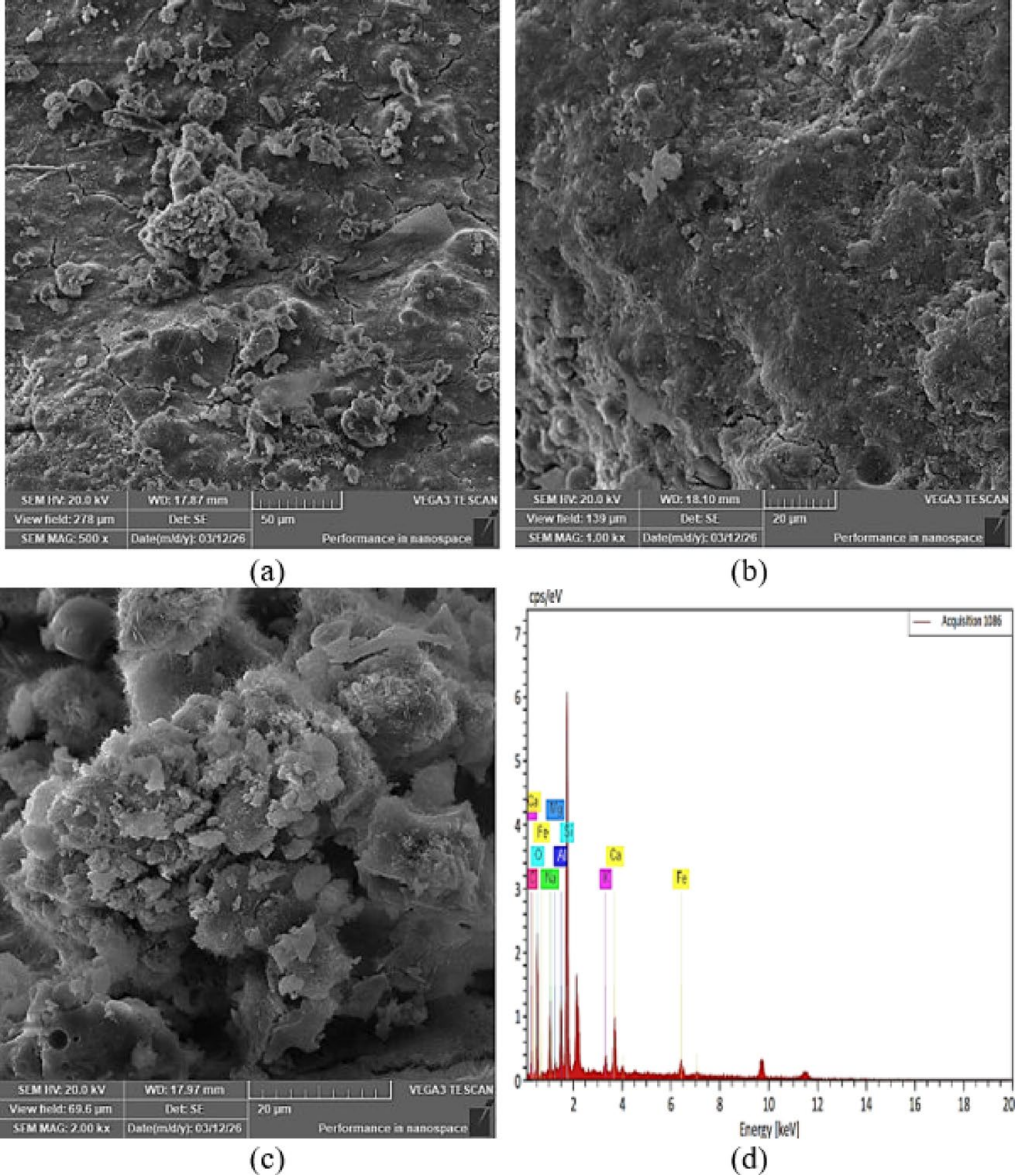
SEM–EDX analysis of the C50 mixture (50% SCBA replacement, 16 M) NaOH) at ambient temperature.

To visually capture the degradation at higher dosages, Fig. 36 presents the SEM–EDX results of the C50 mixture (Fig. 36a and d). At this 50% replacement threshold, the microstructure appeared significantly more heterogeneous and porous, accompanied by a high Si/Al ratio of approximately 4.55 and a reduced compressive strength of 17.22 MPa. Compared with the C25 system, this higher SCBA loading severely undermined the matrix continuity and restricted the full development of a well-integrated geopolymeric binder phase.

Further increasing the SCBA content to 75% (C75) intensified the macro-pore formation and structural discontinuities, while the Si/Al ratio climbed sharply to approximately 7.61, causing the compressive strength to decline to 8.41 MPa. This dramatic increase in the Si/Al ratio is associated with a relative reduction in aluminum-containing reaction products rather than an enhancement in geopolymerization quality. Consequently, the lower availability of reactive aluminosilicate phases and the subsequent reduction in matrix cohesiveness led to a progressive deterioration in overall mechanical performance as the SCBA replacement level advanced.

In summary, the microstructural screening confirms that escalating the SCBA replacement beyond 25% induces a chemical imbalance marked by an excessive silica surplus that diminishes reactive aluminum capture. This induces a physical transition from a continuous, load-bearing binder to a highly porous and unbonded microstructure, microstructurally verifying why higher ash replacement limits mechanical efficiency.

## Cost analysis and sustainability assessment

As presented in Table 10, increasing the SCBA content resulted in a substantial and systematic reduction in the estimated production cost of the GPC mixtures. The estimated raw-material cost decreased from 31,865.15 L.E/m³ for Control A to 9,065.15 L.E/m³ for A95, representing an estimated cost reduction of approximately 71.5% under the adopted assumptions. Similar trends were observed across the B and C series. This reduction was primarily attributed to the replacement of comparatively high-cost fly ash with SCBA.

SCBA was assigned a zero acquisition cost at the factory source because it is an agricultural by-product generated during the routine operation of the sugar industry. The costs associated with bagasse drying and combustion were not allocated to SCBA because these processes form part of the factory’s normal energy-production operations. However, the costs of collection, transportation, sieving, and handling were not quantified because they depend on site-specific factors, including transportation distance, processing scale, logistics, and local labor costs. Therefore, the present assessment represents a comparative raw-material cost analysis rather than a complete life-cycle cost analysis. Excluding these site-specific costs may overestimate the reported savings, particularly for mixtures containing high SCBA contents. Accordingly, the calculated savings should be considered conditional estimates and possible upper-bound values. A sensitivity analysis was not performed because reliable site-specific cost data were unavailable and should be considered in future investigations.

No formal multi-criteria weighting or optimization framework was applied in this study. Therefore, the mixtures were compared qualitatively in terms of engineering performance, durability, estimated production cost, and SCBA utilization.

Among the SCBA-containing mixtures, B25, incorporating 25% SCBA and activated with 14 M NaOH, exhibited the highest mechanical performance and favorable durability characteristics. Its estimated raw-material cost was 26,048.01 L.E/m³, corresponding to a conditional cost reduction of approximately 18.7% relative to Control B under the adopted assumptions. Therefore, B25 is presented as a practical compromise between engineering performance, cost reduction, and SCBA utilization, rather than as an absolute optimum mixture. Control B remained the best-performing mixture when engineering properties alone were considered.

Increasing the SCBA content beyond 25% produced further cost reductions; however, these mixtures exhibited substantial decreases in mechanical strength and increased sorptivity, indicating greater porosity and limiting their suitability for structural applications. Nevertheless, mixtures containing higher SCBA contents, such as B95 and C95, may be considered for selected non-structural applications where lower strength requirements are acceptable.

Beyond the economic benefits, SCBA utilization may provide environmental advantages through the beneficial use of agricultural waste and the reduction of disposal and landfilling requirements. Previous life-cycle assessment studies have reported that geopolymer binders can exhibit lower embodied carbon emissions and environmental impacts than conventional Portland cement-based systems, although the environmental contribution of alkaline activators should also be considered^49–54^. Furthermore, replacing fly ash with SCBA promotes waste valorization and resource circularity. However, a complete environmental assessment of the investigated mixtures was beyond the scope of the present study and should be addressed through a future life-cycle assessment.

**Table 10:** Estimated raw-material cost of the investigated geopolymer concrete mixtures (L.E/m³).

Mix ID		SF (L.E)	FA (L.E)	SCBA* (L.E)	Na_2_SiO_3_ (L.E)	NaOH Flakes(L.E)	S.*P* (L.E)	Sand (L.E)	Dolomite (L.E)	Total cost (L.E)
Control A		1000	22,800	-	3214.25	1462.898	500	688	2200	31865.15
A25		1000	16,800	-	3214.25	1462.898	500	688	2200	25865.15
A50		1000	10,800	-	3214.25	1462.898	500	688	2200	19865.15
A75		1000	4800	-	3214.25	1462.898	500	688	2200	13865.15
A95		1000	0	-	3214.25	1462.898	500	688	2200	9065.148
Control B		1000	22,800	-	3214.25	1645.76	500	688	2200	32048.01
B25		1000	16,800	-	3214.25	1645.76	500	688	2200	26048.01
B50		1000	10,800	-	3214.25	1645.76	500	688	2200	20048.01
B75		1000	4800	-	3214.25	1645.76	500	688	2200	14048.01
B95		1000	0	-	3214.25	1645.76	500	688	2200	9248.01
Control C		1000	22,800	-	3214.25	1834.994	500	688	2200	32237.24
C25		1000	16,800	-	3214.25	1834.994	500	688	2200	26237.24
C50		1000	10,800	-	3214.25	1834.994	500	688	2200	20237.24
C75		1000	4800	-	3214.25	1834.994	500	688	2200	14237.24
C95		1000	0	-	3214.25	1834.994	500	688	2200	9437.244

*SCBA was assigned a zero acquisition cost at the factory source. Collection, transportation, sieving, and handling costs were excluded because they are site-specific and were not available for the present analysis.

## Conclusions

This study evaluated the feasibility of utilizing sugarcane bagasse ash (SCBA) as a high-volume replacement for fly ash in geopolymer concrete under different alkaline activator molarities. Based on the experimental findings, the following conclusions can be drawn:

- SCBA can be successfully incorporated as a sustainable geopolymer precursor; however, its replacement level significantly influences the microstructural development and overall performance of the geopolymer matrix.
- Increasing the SCBA content reduced workability, density, and mechanical performance due to the porous nature of SCBA and the lower availability of reactive aluminosilicate phases, which limited geopolymerization efficiency and matrix continuity. Specifically, escalating the SCBA replacement beyond 25% induced a chemical imbalance marked by an excessive silica surplus and an aluminum deficit.
- The optimum performance was achieved by mixture B25 (25% SCBA and 14 M NaOH), which provided the most favorable balance between strength, durability, and microstructural integrity. SEM observations confirmed the formation of a relatively dense and homogeneous geopolymeric matrix characterized by a distinct layered, continuous flaky morphology and an exceptional reduction in capillary porosity compared with mixtures containing higher SCBA contents.
- Alkaline activator molarity played a critical role in geopolymer development. Increasing the molarity from 12 M to 14 M enhanced the dissolution of reactive species (achieving an optimal Si/Al ratio of 2.21 for the baseline system) and improved matrix formation. Conversely, further increasing the activator concentration beyond the optimum threshold to 16 M induced a rapid, non-uniform gel precipitation, leading to localized structural heterogeneity and reduced mechanical performance.
- Exposure to initial thermal stages between 100 °C and 200 °C induced noticeable compressive strength gains ranging from 12.92% to 39.30% due to continued geopolymerization and moisture removal. In contrast, temperatures at and above 300 °C represented a critical thermal threshold that initiated severe microstructural deterioration, extensive matrix dehydration, thermal microcrack propagation, and localized matrix loosening, resulting in a progressive and accelerated strength loss.
- Mixtures containing higher SCBA contents exhibited improved thermal stability and lower strength degradation at elevated temperatures, indicating the potential suitability of SCBA-based geopolymer concrete for applications requiring enhanced thermal resistance.
- SEM–EDX analysis demonstrated that changes in microstructure and the Si/Al ratio strongly influenced mechanical behavior. Dense and continuous aluminosilicate networks were associated with superior performance, whereas high porosity and microstructural discontinuities resulted in lower strength and durability. Importantly, the findings critically demonstrate that the physical microstructural condition of the matrix (crack kinetics and pore connectivity), rather than the chemical Si/Al ratio alone, governs the residual mechanical performance after severe thermal exposure.
- From an economic and sustainability perspective, increasing SCBA replacement reduced the overall material cost because SCBA is an agricultural by-product whose cost is primarily limited to transportation. Therefore, SCBA utilization contributes to waste valorization, resource conservation, and the development of more sustainable geopolymer construction materials.

## Recommendations

- Examine ways to enhance geopolymer concrete’s qualities at high SCBA replacement levels. Prioritize strengthening, decreasing porosity, and increasing durability.
- Examine the application of various fiber kinds to enhance mechanical performance. Examine how they affect behavior at high temperatures, tensile strength, compressive strength, and crack resistance.
- Assess long-term resilience in aggressive environments including exposure to sulphate and chloride.
- To improve reactivity, investigate SCBA pre-treatment techniques including grinding or thermal activation.
- Investigate treated alum in high SCBA mixes to determine its impact on strength, durability, and sulphate impact.to enhance Si/AL ratio.
- Examine the field performance and widespread use of SCBA-based geopolymer concrete.
- Examine the production of lightweight geopolymer concrete with a high SCBA content for non-structural uses using lightweight aggregates. Analyze durability, strength, density, and thermal insulation.
- Make detailed life-cycle assessment (LCA) and quantitative CO₂ analysis for SCBA based geopolymer concrete.
- Future studies should quantify the actual unburned carbon and volatile contents of SCBA using thermogravimetric analysis and direct carbon measurements. The effects of batch-to-batch variability and high LOI on alkaline activator demand, superplasticizer adsorption, workability, reaction kinetics, and mechanical performance should also be investigated. In addition, suitable pretreatment methods, such as controlled reburning, washing, or further grinding, may be evaluated to reduce LOI and improve the consistency and reactivity of SCBA.
- Future work should include a comprehensive life-cycle cost and environmental assessment, together with a sensitivity analysis considering SCBA collection, transportation, sieving, processing, handling, transportation distance, and local labor and energy costs.

## Data Availability

All data generated or analyzed during this study are included in this published article.

## Abbreviations

Alk.: Alkaline solution
C-A-S-H: Calcium aluminosilicate hydrate
EDX: Energy dispersive X–ray analysis
FA: Fly ash
F_ch_: Schmidt hammer estimated strengths
F_cu_: Compressive strengths
GGBS: Ground granulated blast furnace slag
GPC: Geopolymer concrete
L.O.I: Loss of Ignition
N-A-S-H: Sodium aluminosilicate hydrate
Na_2_SiO_3_: Aqueous sodium silicate solution
NaOH: Sodium hydroxide
OPC: Ordinary portland cement
SCBA: Sugarcane bagasse ash
SEM: Scanning electron microscopy
SH: Schmidt hammer
XRD: X–ray diffraction
